# The bacterial ESCRT-III PspA rods thin lipid tubules and increase membrane curvature through helix α0 interactions

**DOI:** 10.1073/pnas.2506286122

**Published:** 2025-08-04

**Authors:** Esther Hudina, Stephan Schott-Verdugo, Benedikt Junglas, Mirka Kutzner, Ilona Ritter, Nadja Hellmann, Dirk Schneider, Holger Gohlke, Carsten Sachse

**Affiliations:** ^a^Ernst-Ruska Centre for Microscopy and Spectroscopy with Electrons, ER-C-3: Structural Biology, Forschungszentrum Jülich, Jülich 52425, Germany; ^b^Department of Biology, Heinrich Heine University Düsseldorf, Düsseldorf 40225, Germany; ^c^Institute of Bio- and Geosciences, IBG-4: Bioinformatics, Forschungszentrum Jülich, Jülich 40225, Germany; ^d^Department of Chemistry, Biochemistry, Johannes Gutenberg University Mainz, Mainz 55128, Germany; ^e^Institute of Molecular Physiology, Johannes Gutenberg University Mainz, Mainz 55128, Germany; ^f^Institute for Pharmaceutical and Medicinal Chemistry, Faculty of Mathematics and Natural Sciences, Heinrich Heine University Düsseldorf, Düsseldorf 40225, Germany

**Keywords:** ESCRT, cryo-EM, molecular dynamics

## Abstract

The phage shock protein A (PspA), a bacterial member of the endosomal sorting complexes required for transport (ESCRT)-III superfamily, forms rod-shaped helical assemblies that internalize membrane tubules. The N-terminal helix α0 of PspA (and other ESCRT-III members) has been suggested to act as a membrane anchor; the detailed mechanism, however, of how it binds to membranes and eventually triggers membrane fusion and/or fission events remains unclear. By solving a total of 15 cryoelectron microscopy (cryo-EM) structures of PspA and a truncation lacking the N-terminal helix α0 in the presence of *Escherichia coli* polar lipid membranes, we show in molecular detail how PspA interacts with and remodels membranes: Binding of the N-terminal helix α0 in the outer tubular membrane leaflet induces membrane curvature, supporting membrane tubulation by PspA. Detailed molecular dynamics simulations and free energy computations of interactions between the helix α0 and negatively charged membranes suggest a compensating mechanism between helix-membrane interactions and the energy contributions required for membrane bending. The energetic considerations are in line with the membrane structures observed in the cryo-EM images of tubulated membrane vesicles, fragmented vesicles inside tapered PspA rods, and shedded vesicles emerging at the thinner PspA rod ends. Our results provide insights into the molecular determinants and a potential mechanism of vesicular membrane remodeling mediated by a member of the ESCRT-III superfamily.

## Significance

The endosomal sorting complexes required for transport (ESCRT) form an evolutionary protein superfamily involved in many cellular membrane remodeling activities. The family has recently been extended by archaeal and bacterial members in addition to eukaryotic proteins. The bacterial member PspA is part of *the phage shock protein* (psp) response that maintains structural integrity of membranes under stress conditions such as temperature and infection. While PspA was shown to form helical superstructures capable of remodeling membranes, the mechanisms of lipid interaction and membrane deformation remained open. Here, we show that rods of bacterial ESCRT-III protein PspA internalize and thin membrane tubules by overcoming the required bending energy through progressive membrane binding of the N-terminal helix in the PspA assembly.

An intact inner membrane is essential for bacterial cell viability, but stressors such as temperature, osmolarity, organic solvents (e.g., ethanol), or phage infections can destabilize the integrity of membranes. To protect the inner membrane, many bacteria activate the bacterial phage shock protein (Psp) response (1–4). So far, the Psp system is best studied in *Escherichia coli* (*E. coli*) where the components of the Psp system are encoded by the *pspF-pspABCDE* operon. Here, the proteins PspF, PspA, PspB, and PspC form the Psp core elements (5–7). Components of the Psp system have also been identified in other bacteria, cyanobacteria, archaea, and chloroplasts (8), although strict conservation only exists for PspA and PspC (8–10). However, the Psp-network architecture appears to be more complex than previously thought, showing a large unexpected diversity in the distribution and occurrence of Psp components across bacterial and archaeal species (10).

The main effector of the Psp system is PspA, a 25 kDa protein consisting of six α-helices connected by short loops and an elongated hairpin, where the N-terminal helix α0 of the protein remains unfolded in the absence of membranes (11, 12). Proteins of the PspA family (i.e., PspA, Vipp1, and LiaH) form MDa-sized homo-oligomeric assemblies such as carpets, rings, and rods with helical symmetry (11, 13–17). Interestingly, PspA is related to eukaryotic and archaeal endosomal sorting complexes required for transport (ESCRT)-III proteins and has a similar structure (11, 15, 18, 19). Thus, proteins of the PspA family are considered bacterial ESCRT-III proteins. Like their bacterial counterparts, eukaryotic ESCRT-III proteins form homo- and hetero-oligomeric assemblies including sheets, strings, rings, filaments, tubules, domes, coils and spirals (20–23). Although the polymeric assemblies of (bacterial, archaeal, and eukaryotic) ESCRT-III proteins come in very different shapes, their monomer structures share the same architecture, and the assemblies also share a similar motif of α5 being packed perpendicularly against the hairpin of α1+2 (11, 24). Also common to all ESCRT-III proteins is their association with membrane remodeling processes. The ESCRT system in eukaryotes assumes critical roles in many cellular processes, including nuclear envelope sealing (25), plasma membrane repair, lysosomal protein degradation (26), retroviral budding, and the multivesicular body (MVB) pathway (27). While the basic topology of the budding process is directed away from the cytosol (28), the involved membrane geometries can differ. In the first case, ESCRT-III polymers are required to assemble inside of membrane neck structures to negatively curved membranes as observed in vitro and in vivo (29–31). In the second case, ESCRT-III proteins were found to bind to the outside of a membrane tube of positive membrane curvature (21, 23, 32, 33).

How PspA is involved in maintaining the bacterial membrane is so far only poorly understood. It has been shown that PspA binds to negatively charged membranes by the amphipathic N-terminal helix α0 and is capable of membrane remodeling by fusion and fission processes (11, 34, 35). Like Vipp1, a close relative of PspA found in cyanobacteria and chloroplasts, PspA has been suggested to passively protect membranes by forming a protective carpet on the membrane that reduces proton leakage, and/or performing active membrane repair by excising damaged membrane areas and potentially sealing them by fusion with intact membranes (11, 14, 36, 37). However, the molecular details of these processes remained unclear.

We have analyzed the details of PspA–membrane interactions of the thus far unresolved N-terminal helix α0 of PspA of the cyanobacterium *Synechocystis* sp. PCC6803 (form here on PspA). By solving a total of 15 PspA rod cryo-EM structures in the presence of *E. coli* polar lipid (EPL) membranes combined with biophysical and computational methods, we elucidated interactions between PspA and membranes on a molecular level and show that the N-terminal helix α0, located in the lumen of PspA rods, is critical for membrane tubulation: By inserting helix α0 partly into the outer membrane leaflet, PspA generates highly curved EPL membrane tubules. Although the PspA mutant lacking helix α0 shows no structural alterations in the helical rod structure, it can no longer tubulate EPL membranes. Molecular dynamics (MD) simulations and free energy computations suggest a potential orientation of helix α0 on the membrane surface, revealing essential amino acid residues of the N-terminal helix involved in the membrane interaction and providing insights into a compensating mechanism between helix/membrane binding vs. the energy contributions required for membrane bending. In the cryo-EM images of PspA rods with internalized membranes multiple vesicular structures are visible within a single rod, illustrating a potential membrane remodeling pathway. These results indicate how PspA rods assemble on the membrane surface and, through molecular interactions, can tubulate and thin a lipid bilayer within the rod’s lumen until shedded vesicles emerge.

## Results

### Full-Atom Simulation of Helix α0 with a Lipid Bilayer.

**Fig. 1.**
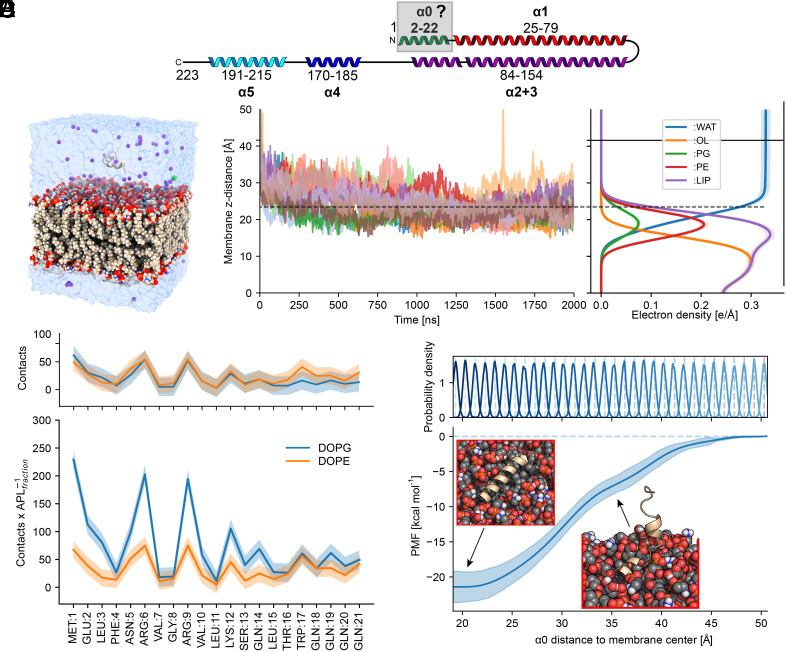
Binding of peptide α0 to a DOPE:DOPG lipid bilayer in MD simulations. (*A*) Secondary structure topology plot of the PspA ESCRT-III fold (α0 green with gray focus box, α1 red, α2+3 violet, α4 blue, α5 cyan). (*B*) Starting configuration for MD simulations, helix α0 located ~25 Å above a DOPE:DOPG (3:1) lipid bilayer, water, and K^+^ as counterions. (*C*) α0 binds spontaneously to the membrane surface in less than 100 ns, as expected for amphipathic peptides. *Left*: distance of the center of mass of the peptide along the membrane normal. *Right*: electron density profile, showing a monolayer arrangement with water, headgroup, and inner membrane regions. PG, phosphatidylglycerol; PE, phosphatidylethanolamine; OL oleic acid tail; WAT, water; LIP, sum of all lipid densities. The dashed line indicates the region of the water–membrane interface. (*D*) The average number of contacts of peptide residues with the lipid head groups shows that M1, R6, R9, and K12, all positively charged, interact more frequently with lipid headgroups than the other amino acids (*Top*). Scaling the number of contacts with the area per lipid for DOPG and DOPE to consider the membrane composition reveals that interactions with negatively charged lipids (DOPG) are more frequent (*Bottom*). (*E*) From umbrella sampling simulations, using the distance to the membrane center along the normal as reaction coordinate, a free energy profile (PMF) for peptide (un)binding was computed. The shaded area shows the SD using the unbound state as a reference (*Materials and Methods*). The profile has a minimum at the membrane surface (~20 Å, *C Right*), with a depth of ~−20 kcal mol^−1^ and increases with a slope change between 30 and 35 Å until a distance of 45 Å is reached. *Insets* show the last structures from umbrella sampling windows 2 and 17 (restrained at 20.3 and 36.3 Å, respectively).

To date, the mechanism of PspA interaction with membranes has not been fully characterized, as the full-length PspA cryo-EM structure did not contain density for helix α0 (11). Therefore, we performed biophysical characterization by circular dichroism spectroscopy and tryptophan fluorescence, confirming that the isolated amphipathic PspA-α0 peptide binds to membrane surfaces while forming an α-helical structure (*SI Appendix*, *Supporting Text*). To investigate at the atomistic level how the PspA-α0 peptide interacts with the lipid membrane (Fig. 1*A*), we performed MD simulations of the peptide interacting with a DOPE:DOPG (3:1) membrane. Although initially located 25 Å above the membrane surface (Fig. 1*B*), the peptide bound to the membrane surface in less than 100 ns of simulation time in all 12 replicas while adopting a mainly α-helical secondary structure (Fig. 1*C* and *SI Appendix*, Fig. S2*A*). The peptide formed contacts with the membrane through M1, R6, R9, and, less pronounced, K12 (Fig. 1*D*), in agreement with the biophysical measurements supporting the critical role of the positively charged residues R6 and R9 for binding the negatively charged headgroups of the membrane surface. After 2 µs of unbiased MD simulations, the bound peptide was pulled away from the membrane along the membrane normal using adaptive steered MD simulations, until the peptide reached an unbound state with a distance of ~50 Å from the membrane center. That way, starting structures for umbrella sampling (US) simulations were generated and a free energy profile [potential of mean force (PMF)] of peptide (un)binding was computed. Initially, the PMF indicated strong binding of peptide α0 to the membrane surface (at the water–membrane interface ~20 Å). Upon unbinding, the energy rises steadily until it reaches a plateau beyond 45 Å to the membrane center (Fig. 1*E*). At the lowest energy, the related US structures had a continuous α-helix conformation, with an orientation at the membrane surface constrained by the membrane interactions. With increasing energy, α0 split up into two α-helical segments introducing a kink at G8 and, at the highest energy, a flexible loop (*SI Appendix*, Fig. S2 *B*–*F*). Integration of the PMF showed that the major energetic contribution occurred at a distance to the membrane center below 30 Å (*SI Appendix*, Fig. S2*G*). The PMF at distances of 30 to 35 Å to the membrane center marked the detachment of the C-terminal region of the peptide, while the N-terminal region maintains intramolecular hydrogen bonds between E2 and R6/R9, stabilizing the α-helix and kink topology (*SI Appendix*, Fig. S2*H*) (38). The integration of the PMF, considering the loss of configurational and conformational entropy upon membrane-binding of the peptide (*Materials and Methods*), yielded a binding free energy of ∆*G*^0^ = −6.32 ± 2.07 kcal mol^−1^ for binding of the isolated helix α0 to the model membrane, which is in agreement with the experimentally determined ∆*G*^0^ values (*SI Appendix*, *Supporting Text*). Together, the MD peptide simulations support the biophysical characterization obtained with the isolated helix suggesting binding of the PspA N terminus to a DOPG:DOPE membrane by forming an amphipathic α-helix next to the lipid headgroups.

### Cryo-EM Reveals Dilated Rods Engulfing Membranes through Helix α0 Interactions.

**Fig. 2.**
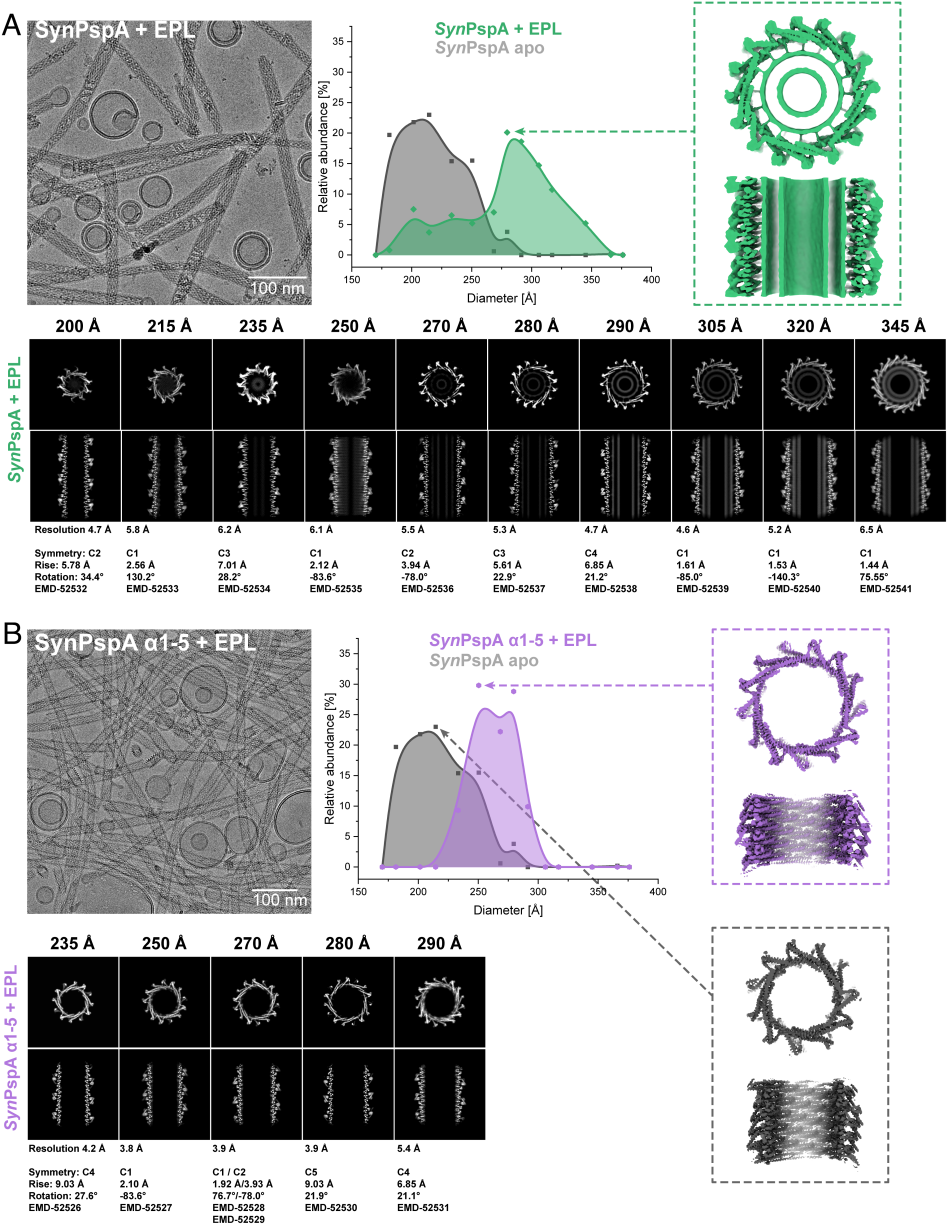
PspA rod diameter distribution and corresponding cryo-EM structures in the presence of membranes. Example micrographs (*Left*), PspA rod diameter distributions (center) and cryo-EM density with top and sliced side view (*Right*) for (*A*): PspA+EPL (green) and (*B*): PspA α1-5+EPL (violet), based on the relative occurrence of rod segments with a certain diameter. Highlighted structures (*Right*) show details of the most abundant diameter for each sample [PspA+EPL (green), PspA α1-5+EPL (violet), PspA *apo* (gray)]. A gallery of PspA rod cryo-EM structures (*Bottom*) with cross-sectional top views or *z*-slices (*Top* row) and cross-sectional side views or *xy*-slices (*Bottom* row) is shown, with information on resolution and symmetry below.

To understand the mechanism of membrane remodeling by PspA, we examined cryo-EM structures of PspA in the presence of EPL membranes. For our analyses, we refolded PspA in the presence of 50 nm sized small unilamellar EPL vesicles, similar to a recently described protocol (39, 40). In the EPL sample, we detected a large number of PspA rods showing varying diameters within one rod, revealing the lack of a persistent structure along the rods (Fig. 2*A*). In the presence of EPL membranes, the rods had a wider diameter and a broader diameter distribution (200 to 345 Å) in comparison with the diameter values and distribution of the previously analyzed *apo* protein (180 to 290 Å) (39). By segmenting the rods of the EPL-PspA sample and sorting the segments according to diameter through classification, we determined a total of 10 cryo-EM structures with resolutions ranging from 4.7 to 6.5 Å (*SI Appendix*, Fig. S3 *A* and *B* and Table S1). While the imposed helical symmetry parameters suggested no architectural relationship between the PspA rods, helical lattice plots revealed that the left-handed helical rung corresponding to layer line ~110 Å in the power spectrum increased in Bessel order from n = 10, 11, 12…18 with increasing diameters of 200, 215, 235…345 Å, respectively (*SI Appendix*, Fig. S4 and Table S2). Upon insertion of an additional subunit into the helical rung, the assembly widens in discrete steps of around 20 Å up to a diameter of 345 Å. The rods with diameters larger than 280 Å were of particular interest as they showed enclosed double-layered lipid density in the cross-sectional top and side views in addition to the expected PspA protein densities, whereas rods of 270 Å diameter contained only partial lipid density and smaller diameters showed no density in the cross-section views. Due to the critical role of helix α0 in membrane interaction, we also prepared and refolded an N-terminally truncated PspA including helices α1-5 in the presence of EPL liposomes. For the truncated PspA α1-5 sample, the total diameter distribution was narrower and ranged from 235 to 290 Å, with two maxima at 255 Å and 275 Å that were closer but not identical to the *apo* distribution of WT PspA (Fig. 2*B*). In the PspA α1-5 + EPL sample, we determined a total of five cryo-EM structures with resolutions ranging from 3.8 to 5.4 Å supported by visible side-chain details (*SI Appendix*, Fig. S3 *C* and *D* and Table S3). None of these structures contained any additional lipid density in the rod lumen. Recent work has shown that PspA is an atypical ATPase with a low hydrolysis rate (39) while this ATPase activity has been suggested to relate to PspA’s membrane remodeling activity. Using an ADP-Glo assay, we confirmed the reported adenosine triphosphate (ATP) hydrolysis by the WT protein with a rate of 3 h^−1^ (*SI Appendix*, Fig. S3*E*). Interestingly, when the protein was reconstituted in the presence of membranes, the ATPase activity was increased by ~210%, while the activity of PspA α1-5 was not affected in the presence of membranes. The combined analyses of the isolated PspA helix α0 and the PspA cryo-EM structures suggest that for membrane engulfment into PspA rods helix α0 as well as the formation of rods with diameters beyond 280 Å are required and that PspA hydrolysis of ATP is boosted by the presence of membranes.

**Fig. 3.**
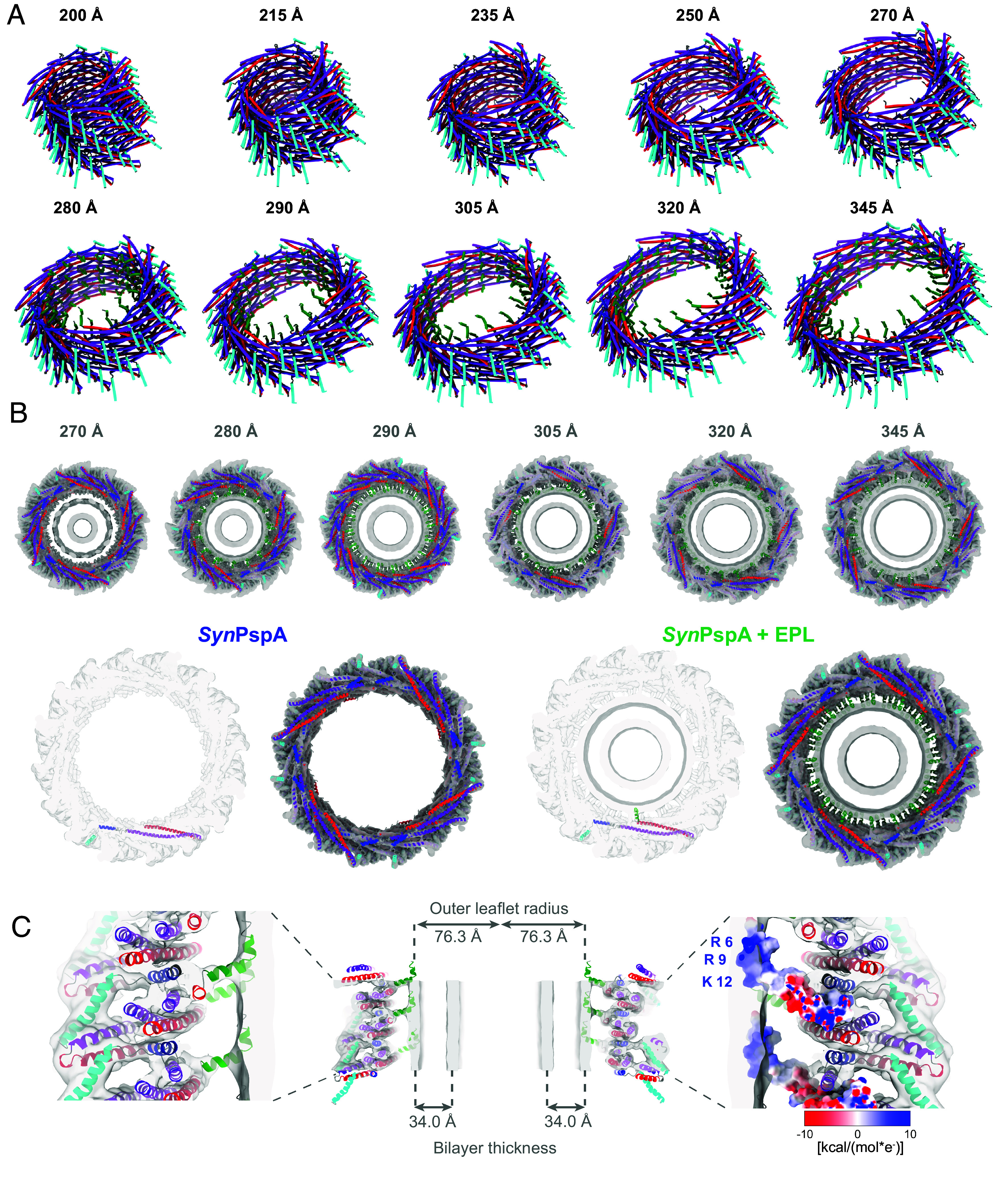
Membrane interaction of PspA rods. (*A*) A total of 10 ribbon models of helical assemblies of PspA+EPL with diameters from 200 to 345 Å (helix α0 green, α1 red, α2+3 violet, α4 blue, α5 cyan). (*B*) Top row: Gallery of PspA+EPL rods containing intralumenal lipid density (Top views of the cryo-EM density maps with the atomic model of the respective polymer structure, helix α0 green, α1 red, α2+3 violet, α4 blue, α5 cyan). *Bottom* row: Top view of the cryo-EM density maps with the atomic model of the 290 Å PspA rods from PspA (*Left*) and PspA+EPL (*Right*); helix α0 green, α1 red, α2+3 violet, α4 blue, α5 cyan. (*C*) Sliced side view of a 290 Å PspA+EPL rod. *Left*: Vertical sliced side view of the cryo-EM density map with the atomic model. *Center*: Complete vertical density section with atomic model including measurements for the outer leaflet radius and the bilayer thickness of the engulfed lipid tube. *Right*: Sliced side view of the cryo-EM density map with the Coulomb electrostatic potential of the monomer. R6, R9, K12: Positively charged amino acids of helix α0 partially inserted in the headgroup region of the bilayer.

To investigate the details of membrane interaction, we refined 10 atomic models of the different rod diameter assemblies using the determined cryo-EM maps based on the previously published structure of PspA (PDB:7ABK). When comparing them with the previous *apo* structures (11, 39), we observed that the structures of the EPL sample were identical to the *apo* structures with the same diameters, including their respective helical symmetry (Fig. 3*A* and *SI Appendix*, Fig. S5*A*). For the α1-5 PspA variant, we found only five rod diameters (235, 250, 270, 280, and 290 Å), whereas two different symmetries (C1 and C2) could be identified for the diameter of 270 Å (*SI Appendix*, Fig. S5*B*). In the WT structures, we were able to clearly assign EM density to helices α1-5 whereas continuous density corresponding the helix α0 could only be observed in diameters 280, 290, 305, 320, and 345 Å and not for the 270 Å diameter (Fig. 3*B*). Helix α0 was found as a well-defined density stalk that connected the core PspA-fold with the bilayer density, creating a constant distance of ~35 Å between the end of helix α0 and the bilayer center (Fig. 3*C*). This connecting stalk only accommodated half of the α0 helix before merging perpendicularly with the outer leaflet of the bilayer in the rod lumen. The density and the modeled helix α0 structure resembled the intermediately bound structure found in US simulations of isolated helix α0 (Fig. 1 *E*, *Inset*). Therefore, we included the kink at G8 in helix α0, as identified in the US, which allowed the alignment of the positively charged residues R6, R9, and K12 with the negatively charged membrane headgroup region. Thus, the N-terminal part of helix α0 inserts into the lipid bilayer likely promoting positive membrane curvature. When comparing the tubulated EPL membranes inside rods, we found that the bilayer thickness remained constant (34 Å) regardless of the different rod diameters, even when the radius of the rods and thus the outer membrane leaflet radius increased (*SI Appendix*, Fig. S5 *C*–*E*). Together, the PspA cryo-EM structures solved in the presence of membranes revealed structural details of how α0 binds to membranes and lipid interactions contribute to internalizing membrane tubules into the lumen of PspA rods with different diameters.

### Molecular Dynamics Simulations Capture the Contributions of Helix α0 Binding and Membrane Bending Energy.

**Fig. 4.**
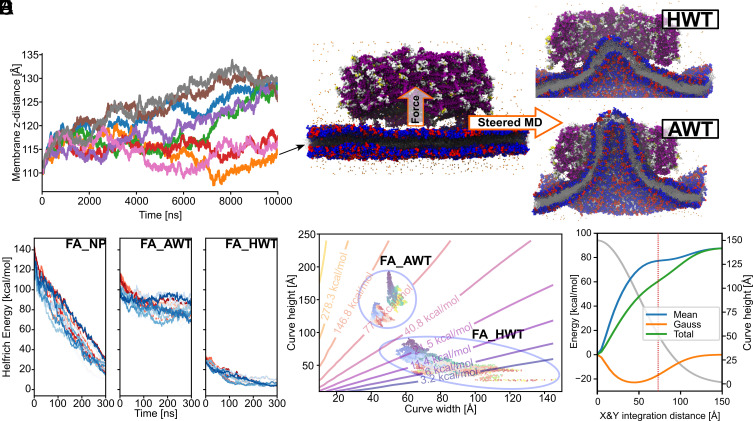
Interactions of the PspA 290 Å diameter assembly with the membrane. (*A*) In coarse-grained molecular dynamics simulations (CG-MD), for three out of eight replicas the center of mass distance of PspA to the membrane center along the membrane normal decreased. (*B*) The final structure with the shortest distance was used as a starting point for steered CG-MD. Two structures were selected to be backmapped to a full-atom representation and further analyzed, one with the membrane HWT and one with the membrane AWT. (*C*) Helfrich energy over the MD simulation time for full-atom no PspA (FA_NP), full-atom AWT (FA_AWT), and full-atom half-way-through (FA_HWT). The shape of the upper (shades of blue) and lower (shades of red) membrane leaflets in the simulations were approximated by 2D Gaussian functions, from where the curvature and the associated Helfrich energy were computed (also refer to *SI Appendix*, Fig. S6*B*). (*D*) Helfrich energy isoenergy contour plot for membrane surfaces represented with different heights and widths as 2D Gaussian functions. The same width was taken for both dimensions. Helfrich energies of snapshots of FA_AWT and FA_HWT trajectories are projected onto the isocontours (blue to yellow, for start-to-finish upper leaflet and light blue to red, for start-to-finish lower leaflet). For a 3D depiction of the energy surface refer to *SI Appendix*, Fig. S7. (*E*) Total, mean, and Gauss bending energy contributions as a function of integration limits in the X/Y plane from the center of the membrane surface. A representative Gaussian function for FA_AWT (see panel *D*) with 150 Å of amplitude and 50 Å of SD in both dimensions was used (cross section in gray, right y-axis). The vertical dashed line shows the calculated inner cavity radius as calculated by HOLE (43) on the cryo-EM structure. The major energy contribution occurs close to the tip of the membrane surface, while increases become smaller as the inner diameter of the protein is reached.

The cryo-EM analysis revealed that only PspA tubes wider than 280 Å contained a clearly resolved membrane tube in their inner lumen (Fig. 2). To investigate the structural dynamics and energetics of a representative 290 Å diameter PspA rod in the presence of a membrane, we performed coarse-grained (CG) simulations with the SIRAH force field (41, 42). Unbiased simulations over 10 µs of eight replicas showed no strong interaction of 160 Å high PspA assemblies with the membrane surface; in only three replicas, the PspA assembly approached the membrane but did not bind to it (Fig. 4*A*). We thus resorted to biased CG simulations to steer the membrane through the center of the PspA rod. For this experiment, the replica that showed in its last frame the shortest distance along the membrane normal between the PspA assembly and the membrane was chosen. Two structures at different pulling points were backmapped to a full-atom (FA) representation: i) where the membrane reached “half-way-through” (HWT) the assembly and ii) where the membrane reached “all-way-through” (AWT), i.e., fully covered the height of the PspA assembly (Fig. 4*B* and *SI Appendix*, Fig. S6*A*). An additional “no-protein control” (NP) based on full-atom AWT, was prepared, for which the protein was replaced by water to evaluate membrane behavior in the absence of PspA. In all eight PspA-containing unbiased MD simulations of 300 ns with a full-atom representation starting from AWT, the membrane tubule remained internalized in the lumen of the PspA rods. In contrast, when starting from HWT, the membrane retracted from the lumen in all eight replicas and became flat. The same retraction was observed for the control simulations of the AWT membrane shape when no PspA was present.

Based on these calculations and observations, we hypothesized that two opposing energetic contributions give rise to the observed differential behavior of the PspA–membrane systems: on the one hand interactions between PspA and the membrane, mediated by helix α0 formation (Fig. 1 and *SI Appendix*, Fig. S1) that favor membrane internalization, and, on the other hand, energetic costs associated with bending a membrane that disfavors membrane internalization (44). Throughout the MD simulations starting from AWT, PspA tightly interacted with the curved membrane surface mainly through residues 1 to 81, with a major contribution from α0 and, in particular, R9 (*SI Appendix*, Fig. S6*B*). As the AWT simulations progressed, the number of α0 helices interacting with the membrane surface increased to 20 to 30 (out of 60) (*SI Appendix*, Fig. S6*C*) whereas in the case of HWT, less than 10 α0 helices interacted with the membrane throughout the MD simulations. Assuming that the PspA–membrane interaction is dominated by each helix α0–membrane interaction, the favorable contribution to membrane internalization is thus at least two times larger in the AWT than in the HWT scenario. In support of this analysis, when we abolished the α0–membrane interaction by removing helix α0 lacking this binding energy contribution (Fig. 2*B*), we did not observe any membrane internalization in the lumen of the PspA (α1-α5) rods in the cryo-EM images.

In order to assess the energy associated with bending a symmetric lipid bilayer, we used a Helfrich Hamiltonian that describes the energy in terms of the mean and Gauss curvatures at a given surface area segment (*Materials and Methods*). To obtain a smooth representation of the membrane curvature caused by PspA adhesions, while removing local curvature effects due to fluctuations of the membrane surface, the shape of the leaflet surfaces was approximated by a 2D Gaussian (“bell curve”) function. The Helfrich bending energy computed for the “no-protein-control” and HWT MD simulations approached zero over time (Fig. 4*C*), as expected from the decreasing curvature of the retracting membrane. In contrast, for the AWT simulation, the Helfrich energy decreased over the first half of the MD simulations but then plateaued after approximately 150 ns at values between 70 and 90 kcal mol^−1^ for the upper and lower leaflets, in line with the membrane tubule remaining internalized. Notably, in this case, the total Helfrich energy of ~160 kcal mol^−1^ is within the range of the total binding free energy of 20 to 30 helices α0 to the membrane (~125 to ~190 kcal mol^−1^), suggesting that the relaxed AWT simulation was in an equilibrium state. To generalize this analysis, Helfrich energies were computed for membrane surfaces with different heights and widths represented as a 2D Gaussian function and displayed as isoenergetic lines (Fig. 4*D*). The Helfrich energy steeply rose when the curve height increased and when the curve width decreased (*SI Appendix*, Fig. S7). Helfrich energies obtained for snapshots of the AWT and HWT trajectories were projected onto the isoenergetic lines. In both cases, the scenarios started at higher Helfrich energies and relaxed over the simulation time toward membranes with lower heights and larger widths, although AWT isoenergetic lines remained in a region of medium Helfrich energy (Fig. 4*C*). Notably, the AWT line is also located in a region where the Helfrich energy rapidly increased with decreasing membrane widths. The energetic consideration indicates that PspA bending of membranes to narrower diameters is costlier than to wider diameters. The computed bending energies for narrower diameters may explain why PspA rods below a diameter of 280 Å were not found to engulf any membrane in the cryo-EM images: For rods with higher diameters the required bending energy is reduced, while in parallel the number of interacting α0 helices increases, which may explain the experimentally observed shift toward wider diameters (Fig. 2*A*).

Moreover, integrating the membrane surface from the tip of the membrane surface to the rim in a stepwise manner and plotting it against the Helfrich energy reveals that the major Helfrich energy cost occurs next to the membrane-interacting tip indicated by a change in the steep slope (Fig. 4*E*) (*Materials and Methods*). While the mean curvature component is highly unfavorable in this region, the Gauss curvature component contributes favorably, which has been shown to be a driving component in membrane fission of membrane tubules (see below) (45). Once the initial curvature is induced, the highest energy cost has been overcome, while extending the tubule is less costly and will be sustained by further binding of α0 helices in the lumen of the PspA rod. Overall, the higher bending energy cost for forming high membrane curvature suggests that wider initial diameters of PspA with less curved membranes require a lower cost for initiating membrane tubulation. MD simulations together with bending energy considerations of membrane tubule formation reveal the initially high bending energy costs for inducing high membrane curvature, while successive and additive binding of the N-terminal helix α0 to membranes interactions inside the assembly lumen finally overcome the required bending energy by binding energy gains, resulting in membrane tubulation inside tubular PspA assemblies.

### Visualization of Membrane Structures in Variable Diameter Tubes.

**Fig. 5.**
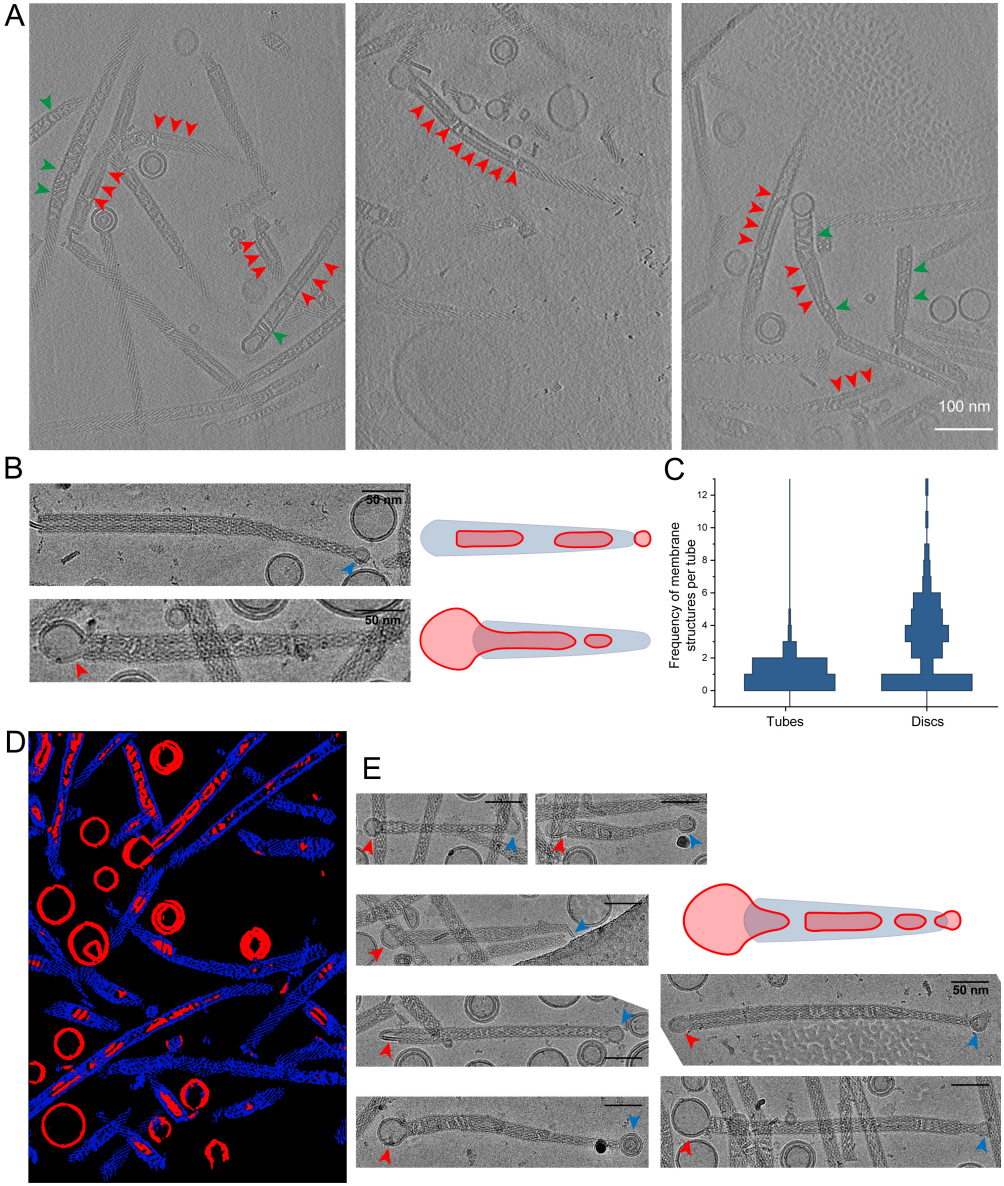
Tomographic analysis of PspA-remodeled membranes. (*A*) Tomogram slices of PspA with EPL membranes. Red arrows: continuously tubulated EPL vesicles within the PspA rod structures. Green arrows: discontinuous membrane discs within the PspA rod structures. (*B*) PspA frequently forms conically shaped rods, with vesicles at the wide as well as the narrow end of the rods. Blue arrow: vesicles near the thinner end of the PspA rod structures do not reach into the lumen of PspA. Red arrow: vesicles near the wider end of the PspA rod structures are tubulated into the lumen of PspA. (*C*) Horizontal histogram plot of membrane tubes and membrane discs located within the PspA rods (n = 125). (*D*) Segmentation of a PspA tomogram with tubulated EPL membranes. Blue: PspA rods. Red: EPL vesicles and tubulated membrane within the PspA rod lumen. (*E*) Red arrows: Beginning of membrane tubulation of EPL vesicles on the thicker side of the PspA rod structures. Blue arrows: vesicles near the thinner end of the PspA rod structures.

Next, we acquired tomograms of the PspA+EPL sample and comprehensively analyzed the PspA ultrastructures and associated membrane structures (Fig. 5*A*). Membranes were found in the form of stand-alone vesicles, rod-attached vesicles, and vesicles internalized in the lumen of PspA rods (*SI Appendix*, Fig. S8*A*). In agreement with the single-particle micrographs of PspA rods, a detailed analysis of the diameter distribution showed that most rods (with or without membrane) vary in diameter along their length, while only a few maintain a constant width (*SI Appendix*, Fig. S8*B*). When we counted vesicles attached to the rod ends, we found 106 (64%) vesicles close to the thinner and 59 (35%) vesicles close to the thicker end typically spanning diameters between 20 and 75 nm (Fig. 5*B*). When we quantified the number of rods containing tubulated membranes inside, 84 out of 249 (34%) were located on the thick end of the rods, the remaining 165 (66%) rods had vesicles centrally incorporated, whereas no vesicles were internalized at the thin end, in line with the above estimated lower Helfrich bending energy that needs to be overcome for wider rods (Fig. 4*D*). Interestingly, only those vesicles attached to the thicker ends showed an internalized lipid bilayer as part of a continuous vesicular bilayer, in agreement with the cryo-EM structures that revealed internal lipid bilayer density for rods with diameters between 280 and 345 Å. Within the lumen of the rods, we identified in some cases continuous membrane tubules, while in other cases discontinuous membrane structures, i.e., PspA rods engulfed multiple tubulated vesicles as separated mini-vesicles, vesicular discs, or less structured lipid assemblies (see Fig. 5*A*, red and green arrows, respectively). One or two tubular vesicles and two to five membrane discs occur in an average rod (Fig. 5*C* and *SI Appendix*, Fig. S8*C*). The segmented three-dimensional tomograms confirmed that these often densely packed membrane structures inside the PspA rods were well separated and not part of a connected membrane network (Fig. 5*D*). When following PspA rods toward smaller diameters, the EPL membrane inside the lumen appears to be constricted concomitantly until separated membrane structures appear. In some cases, we found rods with membrane attached on both ends, first internalizing a continuous lipid bilayer resulting in membrane tubulation at the wider end of the rod, and a small vesicle attached to the thinner end as if it was just going to leave the rod (Fig. 5*E*). Interestingly, the emergence of separated membrane structures in the PspA rod lumen and next to the narrow end of membrane tubules is consistent with a previously discussed pearling mechanism of membrane fission (45).

## Discussion

In this study, we investigated the interaction of the bacterial ESCRT-III protein PspA with membranes. We analyzed the role of helix α0 in membrane interaction by focused biophysical experiments, MD simulations, and resolved it by cryo-EM. In the bacterial ESCRT-III proteins PspA and Vipp1, the N terminus can form a 20 to 30 aa α-helix (helix α0) in the presence of membranes (11, 12, 34). In eukaryotic ESCRT-IIIs, this extension tends to be shorter (3 to 20 aa) and has not been shown to form a structured α-helix to date albeit for instance in the CHMP2A/CHMP3 structure, the corresponding residues are positioned toward the membrane interface (29). Yeast ESCRT-III proteins, i.e., Snf7, Vps24, Vps2, have amphipathic N-terminal extensions that can function as a membrane anchoring domain, mediating the contact of the ESCRT-III polymer to the membrane (46). As shown for PspA and Vipp1, the hydrophobic face and positively charged residues are critical for membrane interaction of α0 [this study and (13, 40, 47)]. For bacterial ESCRT-III α0 helices, the observed conformation and membrane interaction mode appear to differ between PspA and Vipp1: While helix α0 in Vipp1 forms a straight helix that is embedded into the headgroup region of an outer membrane leaflet with its full length (40), helix α0 in the now solved PspA structures is kinked and only the first half of the helix is embedded in the headgroup region of the outer leaflet. Consequently, the distance between the outer membrane leaflet and rod wall also differs between PspA and Vipp1. In Vipp1, the outer leaflet is in contact with the inner ring/rod surface (i.e., α1 and 2/3), while in PspA, a gap of 35 Å between the membrane center and the rod wall does not indicate a direct interaction of α1+2/3 with the membrane. Here, helix α0 serves as a stalk between the protein and the membrane, maintaining a constant distance. These different α0 conformations likely also affect membrane binding of PspA and Vipp1, such that Vipp1 offers a much larger membrane interaction surface (full α0 and α1+2 in Vipp1 vs. only half of α0 in PspA). Furthermore, in Vipp1, helix α0 also interacts with the rod wall via its positively charged residues to stabilize certain assembly types (40). This is not the case in the PspA+EPL structure, where helix α0 does not show interactions with other parts of the protein. Interestingly, the Vipp1 structure showed an assembly type similar to PspA rods, when helix α0 was removed (13, 40, 48).

Furthermore, our ESCRT-III interaction data reveal strong similarities to membrane interactions of epsin and N-BAR proteins. Similarly, an amphipathic N-terminal helix (that is unfolded in the absence of lipids) is embedded into the membrane and serves as a membrane anchor, while the core of the protein serves as a scaffold for polymer assembly and membrane shaping. For example, the N-BAR protein endophilin has been shown to remodel membranes driven by membrane insertion of its amphipathic N-terminal helix, which also results in membrane tubulation (49, 50). Epsin and other ENTH domain proteins are suggested to induce membrane curvature either by wedging of their N-terminal amphipathic helix into the membrane or by molecular crowding (51, 52). The interaction of N-BAR H0 with membranes has been studied extensively by CG and atomistic MD simulations (50), revealing hydrophobic protein moieties that interact with hydrophobic parts of the bilayer. Notably, membrane defects expose hydrophobic parts that are more common in curved membranes. In return, the binding of H0 to membrane defects recruits additional defects, making this process highly cooperative (53). A curvature sensing and generating function has also been suggested for the PspA and Vipp1 α0 helices (35); thus, it is tempting to speculate that PspA follows a similar mechanism for curvature generation as put forward for N-BAR H0. Apart from curvature sensing and curvature generation, CG simulations have also shown that N-BAR H0 is crucial for stabilizing the N-BAR lattice around the tubulated membrane by dimerizing with neighboring H0 helices, thus helping to form contiguous BAR domain strings (50). Although helix α0 is not directly interacting with neighboring α0s in our PspA rod structures, in Vipp1 tubes and rings, neighboring α0 helices directly interact with each other to form contiguous columns at the inside of the polymers that are suggested to stabilize certain assembly types (40).

In order to better understand membrane curvature induction and tubulation, it is particularly important to understand the energy costs necessary for inducing membrane curvature and how this may be provided through protein binding. In the case of PspA, one of the key events required for membrane tubulation likely is primarily mediated by the vertical insertion of the entire amphipathic helix α0 into the headgroup region of the outer bilayer leaflet. As observed in this manuscript by circular dichroism and tryptophan fluorescence spectroscopy and supported by all-atom MD simulations, helix α0 binds to and forms an α-helix upon insertion into the membrane surface. The cryo-EM structures and MD simulations showed that only rods wider than 270 Å internalize and tubulate membranes. As indicated in the simulations, the energy required for membrane bending likely is too high for thinner rods to bend and internalize membrane tubules. Presumably, this is also the reason why we had to perform MD simulations starting with all-way-through rod-internalized membranes for the 290 Å-wide rods used here instead of starting with a rod bound to a flat membrane. The US simulations revealed that the interaction of helix α0 with membrane surfaces has a strong enthalpic component, but is counteracted by a reduction in entropy, resulting in an estimated association free energy ∆*G*^0^ = −6.32 kcal mol^−1^ per helix, a value close to the experimentally determined ∆*G*. As multiple α0 helices inside a PspA rod are involved in membrane binding, multivalency may lead to a superadditive effect as the decrease in translational and rotational entropy from the association of two bodies is reduced after initial binding, which should favor stronger interactions with the membrane surface. Importantly, our data revealed that the interaction of helix α0 with the membrane causes helix α0 to kink at the structure-breaking amino acid G8, which enables a targeted interaction of the positively charged residues (R6, R9, K12) with the negatively charged head groups of the membrane. Notably, in our simulation data helix α0 in the membrane-bound state is initially membrane-bound as a full α-helix, the kink structure observed in an intermediate state explains the kinked structure observed in the cryo-EM fittings.

The electron micrographs and 3D tomograms of the PspA structures solved in the presence of EPL membranes revealed interesting structural features in addition to the averaged PspA structures. The sample included vesicular spherical membrane structures devoid of protein while lipids were commonly found internalized into the lumen of the PspA rods in the form of membrane tubules. In addition to the continuous membrane tubules, discontinuous membrane structures were found inside the lumen of PspA rods. The occurrence of separated membrane structures within PspA rods is often accompanied by a thinning of PspA rod diameters until in some cases they appear to emerge from the thinner rod ends. The structures observed are consistent with the pearling of small vesicles from the tip of membrane tubules that may be a result of spontaneous membrane fission (45). Although the previous studies investigated theoretical models solely based on the shape and the associated energies of the membranes in the absence of proteins, the similarities to our experimental observations in the presence of PspA are noteworthy. Likely, PspA rods provide the environment to cast membranes into the required shapes until they undergo vesicle shedding and subsequently leave the rods.

**Fig. 6.**
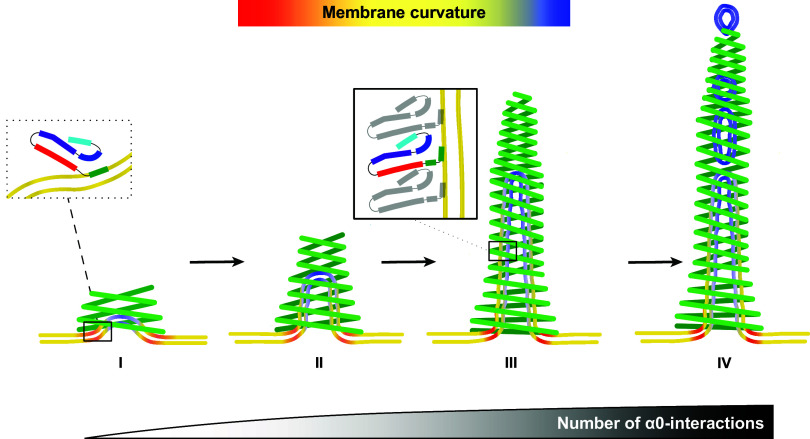
Model of membrane tubulation and vesicle fission mediated by PspA rod structures. I: PspA accumulates on the membrane surface, and the complete helix α0 inserts into the membrane surface (*Inset*). Complexes with a diameter >290 Å are formed and initiate a low-curvature tubulation. II: The initial interactions allow further recruitment of PspA subunits into the complex leading to additional helix α0 interactions with the membrane, which propagate an emerging membrane tubule in the rod lumen. III: Upon extension, the membrane becomes fully tubulated in the lumen of a PspA rod. The membrane has the highest curvature at the tubule tip and base. Extension of the PspA rod with internalized membrane tubes involves partial unbinding of helix α0 by kinking at the structure-breaking amino acid G8 (*Inset*) and as a result, solely the N-terminal part of helix α0 remains membrane-bound. Helix α0 green, α1 red, α2+3 violet, α4 blue, α5 cyan. IV: As observed in tomograms (Fig. 5), at narrow ends of the PspA complex, smaller lipid vesicles emerge from the tubules upon spontaneous fission of the membrane bilayer. The coloring of the bilayer schematically shows the mean curvature of the surfaces upon tubulation and fission; yellow indicates zero mean curvature.

To summarize our observations, we propose the following model for PspA-induced membrane tubulation and vesicle fission: The process of tubulation is initiated by assembling complexes wider than 290 Å (or with small subunits that assemble at the membrane to form a complex wider than 290 Å) on membrane surfaces to minimize the energy barrier required for the induction of membrane curvature. Due to the positively charged residues of the N-terminal region, PspA readily binds to the membrane surface with the entire helix α0. This binding facilitates local curving of the membrane at the tip of an emerging membrane bulge. The energy released by the additive interactions of multiple helices α0 with the membrane compensates for the energy required to initially bend the membrane (Fig. 6*I*, I). Furthermore, the high energy required for initiating membrane bending might also be provided by the energy released due to PspA oligomerization. Oligomerized PspA then stabilizes the membrane curvature; as more α0 helices interact with the membrane, more energy is gained that generates a pulling force leading to the formation of an emerging membrane tubule in the inside of the PspA assembly (Fig. 6, II). Due to the spatially restrained interactions in the lumen of the assembly with the tubulated membrane, helix α0 is partially pulled away from the membrane, which causes a kink in the helix around G8 that contributes to maintaining the membrane interaction of R6, R9, and K12 (Fig. 6, III). Helix α0–membrane interactions in addition to the PspA homo-oligomerization lead to the growth of the tubules, once the bending energy has been overcome during tubule initiation. The distal ends of rods that contain internalized tubulated membrane, i.e., the tips of growing PspA rods, tend to thin toward smaller diameters. Toward the smaller diameters, vesicular membrane structures appear to spontaneously fission from a growing tubule tip, in analogy to the described pearling, until they can leave the rods at the thinner end (Fig. 6, IV) (or they can be ejected at the proximal end, which leads to ILV formation by inward-vesicle budding (see refs. 11 and 39). The described vesicle-shedding pathway through the PspA rod is reminiscent of an ejection process through a molecular nozzle.

While it may be tempting to compare the observed membrane thinning and vesicle shedding to other adenosine/guanosine triphosphate (ATP/GTP)-mediated membrane constriction machines such as dynamin (54), we emphasize that no ATP/GTP was required for the observed membrane remodeling albeit we had previously found that ATP can enhance the efficiency of PspA membrane deformation (39). The membrane thinning and vesicle shedding in the absence of ATP described here is merely a result of the energy gains through helix α0 binding, the directional internalization of lipid tubules toward smaller diameters, and the subsequent increase in negative Gauss curvature resulting in the spontaneous physical separation of small vesicles. The formation of these small vesicles can be explained in terms of a change in curvature energy driven by the Gauss curvature. The increase in membrane curvature favors fission events, as the transition from a single continuous membrane to vesicles is energetically favored by a negative Gauss curvature component (Fig. 4*E*) (55). Eventually, this term leads thin membrane tubules to spontaneously pearl in the absence of additional components (45).

Given the observed variation in PspA rod diameter with respect to membrane deformation, an important question arises regarding the associated dynamics: Do the rods act as a fixed-scaffold assembly or do they taper and widen dynamically? As the static cryo-EM images were plunge-frozen in time, we cannot conclude from the data presented here alone whether the PspA rod diameters maintain a fixed scaffold after tube assembly or whether they taper and widen dynamically. Previous studies revealed that the diameter distribution of PspA rods and the membrane binding capabilities were much smaller when comparing preformed rods with “in situ” polymerized rods after refolding as used here (11, 39). Thus, we can speculate that the rod diameter is predominantly defined during polymer assembly, and the membrane deformation observed here was rather a result of scaffolding and not dynamic tapering and widening. Further experiments with temporal resolution are needed to record rod diameters over time to clarify this question. An additional consideration is that the (re)assembly process may be highly dynamic in the cell when interaction partners such as chaperones or PspF constantly disassemble/assemble PspA rods, effectively leading to a dynamic tapering/widening process as suggested previously (11).

We assume that PspA is involved in an active repair mechanism in the bacterial host cell. Our data now indicate that either PspA rods engulf damaged inner membrane patches by forcing the membrane into high positive curvature eventually leading to extraction of the damaged membrane and membrane resealing, or damaged membranes might be repaired by receiving membrane lipids through PspA-mediated vesicle shedding. Several proposed models of eukaryotic ESCRT-III activities in various biological contexts favor the stabilization of negative membrane curvature eventually, in collaboration with Vps4, membrane cleavage (56) directed away from the cytosol, resulting for instance in budding ILVs away in multivesicular bodies (57, 58). Although the exact mechanistic details remain unclear, several reports provide evidence that ESCRT-III filaments assemble within membrane tubes either in vitro (29, 30) or in vivo (31, 59). In contrast, PspA, Vipp1, archaeal CHMP4-7, and eukaryotic CHMP1B/IST1 have been shown to induce positive membrane curvature and form on the outside of membrane tubes (11, 15, 19, 23). It has also been shown that other eukaryrotic ESCRT-III heteropolymers can bind to membranes from the outside, tubulate, and deform them (33, 58). Nevertheless, eukaryotic ESCRT-III are thought to be more complex as they constitute heteropolymers formed by multiple different ESCRT-III subunits that interact in a certain sequence. How the proposed membrane repair processes for bacterial ESCRT-IIIs compare with the eukaryotic ESCRT-III activities, where vesicles are budded away from the cytosol in the case of canonical ILV formation, remains to be established in future experiments.

## Materials and Methods

### Expression and Purification of PspA.

PspA WT (*orf slr1188*) of *Synechocystis* sp. PCC 6803 and associated mutants [α1-5 (deletion of α0 (aa 1 to 23)] were heterologously expressed in *E. coli* C41 cells in TB medium using a pET50(b) derived plasmid. For purification of PspA and associated mutants under denaturing conditions, cells were resuspended in lysis buffer containing 6 M urea (10 mM Tris-HCl pH 8.0, 300 mM NaCl) supplemented with a protease inhibitor. Cells were lysed in a cell disruptor (TS Constant Cell disruption systems 1.1 KW; Constant Systems). The crude lysate was supplemented with 0.1% (v/v) Triton X-100 and incubated for 30 min at RT. Subsequently, the lysate was cleared by centrifugation for 15 min at 40,000 g. The supernatant was applied on Ni-NTA agarose beads. The Ni-NTA matrix was washed with lysis buffer and lysis buffer with additional 10 to 20 mM imidazole. The protein was eluted from the Ni-NTA with elution buffer (10 mM Tris-HCl pH 8.0, 1,000 mM imidazole, 6 M urea). The fractions containing protein were pooled, concentrated (Amicon Ultra-15 centrifugal filter 10 kDa MWCO), and dialyzed overnight against 10 mM Tris-HCl pH 8.0 (8 °C, 10 kDa MWCO) including three buffer exchanges. Protein concentrations were determined by measuring the absorbance at 280 nm of PspA diluted in 4 M guanidine hydrochloride using the respective molar extinction coefficient calculated by ProtParam (60).

### Liposome Preparation and Membrane Reconstitution.

Chloroform-dissolved EPL extract was purchased from Avanti polar lipids. Lipid films were produced by evaporating the solvent under a gentle stream of nitrogen and vacuum desiccation overnight. The lipid films were rehydrated in 10 mM Tris-HCl pH 8.0 by shaking for 30 min at 37 °C. The resulting liposome solution was subjected to five freeze-thaw cycles, combined with sonication at 37 °C in a bath sonicator. SUVs (small unilamellar vesicles) were generated by extrusion of the liposome solution through a porous polycarbonate filter (50 nm pores). For PspA membrane reconstitution, unfolded PspA (in 6 M Urea) was added to EPL SUVs and incubated at RT for 15 min. Then the mixture was dialyzed overnight against 10 mM Tris-HCl pH 8.0 (8 °C, 10 kDa MWCO) including three buffer exchanges.

### All-Atom MD Simulations of Helix α0 Peptide.

The first 21 residues of the α0 peptide were taken from the first chain of the 290 Å diameter rod structure and capped in the C-terminus with an *N*-methyl group. The latter is required to avoid charge repulsion. The peptide was packed into a water/membrane-bilayer box using PACKMOL-Memgen (61–63) as included in AmberTools 23 (64), using a membrane composition of DOPE:DOPG 3:1 and a minimum distance to the box boundaries of 25 Å, placing the peptide 25 Å above the membrane surface, as estimated by MEMEMBED (62). The packed structure resulted in over 75,000 atoms in a box with dimensions 88.9 Å × 88.9 Å × 112.8 Å. The system was parameterized using the force fields ff14SB (65) for the peptide and LIPID21 (66) for the lipids and the TIP3P water model (67). Hydrogen Mass Repartitioning (68) was applied, allowing to use a timestep of 4 fs.

Twelve replicas were stepwise relaxed, alternating steepest descent/conjugate gradient energy minimizations with a maximum of 20,000 steps each, using the pmemd.MPI implementation. The positions of the membrane were initially restrained during minimization; the final round of minimization was performed without restraints. During the relaxation process, all covalent bonds to hydrogens were constrained with the SHAKE algorithm (69) within the pmemd GPU implementation (70). A direct space nonbonded cutoff of 10 Å was used. The Langevin thermostat with a friction coefficient of 1 ps^−1^ was used, while the pressure, when required, was maintained using a semi-isotropic Berendsen barostat (71) with a relaxation time of 1 ps, coupling the membrane (xy) plane. The system was heated by gradually increasing the temperature from 10 to 100 K for 5 ps under NVT conditions, and from 100 to 300 K for 115 ps under NPT conditions at 1 bar. The thermalization process was continued until 5 ns under NPT conditions were reached, after which production runs of 2 μs length were performed, using the same conditions. Trajectory coordinates were recorded every 200 ps in all cases. For details on adaptive steered MD simulations, umbrella sampling, and free energy estimation from the resulting potential of mean force, refer to the *SI Appendix*, *Supporting Materials and Methods*.

### Coarse-Grain Simulations of a 290 Å Diameter PspA Complex.

As for the helix α0 system, the PspA complex, consisting of 60 protomers, was packed using PACKMOL-Memgen (63), applying options to coarse-grain and parameterize the system with SIRAH (41, 42). The protein was protonated using PDB2PQR (72) with predictions from PROPKA3 (73). The structure was oriented using PPM3 with a flat Gram-negative membrane model (74). As the phosphatidylglycerol (PG) head group is not available in the SIRAH force field, phosphatidylserine (PS) was used instead, which also has a -1 net charge, using a membrane composition of DOPE:DOPS 3:1. At the CG level, we do not expect major differences between both head groups. Considering that the membrane can be curved inside of the protein structure, extra padding was added to the system, ensuring at least 50 Å from the protein to the box limits, resulting in a system with dimensions of 426.6 Å × 426.6 Å × 201.8 Å and over 700,000 CG particles. Eight replicas of the system were prepared and minimized and relaxed in the same way as for helix α0, but totaling 50 ns in the thermalization step. As in the SIRAH AMBER tutorials, the following parameters were used: 20 fs timestep, Langevin thermostat with a collision frequency of 5 ps^−1^ set at 310 K, a semiisotropic Berendsen barostat (71) coupling the xy-plane with a relaxation time of 8 ps and a 12 Å direct space cutoff, using otherwise identical options as for helix α0. Production runs yielded 10 µs per replica.

To obtain structures where the membrane interacts with the center of the PspA complex, the CG replica that showed the lowest distance between the center of mass of the complex and the membrane center after the production runs was selected. Similar to the pulling of helix α0 (see above), AsMD simulations were started, using as reaction coordinate (RC) the distance along the z-axis between the COM of the GC beads (C_α_ atom-equivalent) of the protein complex and the COM of the lipids that have any atom within 290/2 Å in the xy plane to the GC COM. Twenty iterations of 25 ns with eight replicas in parallel were performed, selecting as a restart structure after each iteration the replica with the work closest to the Jarzynski average, pulling from a RC of −117.9 to 7.1 Å (0.25 Å ns^−1^). This results in a trajectory where the membrane fully reaches through the center of the protein complex. For details on the backmapping of the CG structure to an all-atom structure and membrane curvature analysis, refer to the *SI Appendix*, *Supporting Material and Methods*.

### Electron Cryomicroscopy.

PspA grids were prepared by applying 3.5 μL PspA (*SI Appendix*, Table S4) to glow-discharged (PELCO easiGlow Glow Discharger, Ted Pella Inc.) Quantifoil grids (R1.2/1.3 Cu 200 mesh, Electron Microscopy Sciences). The grids were plunge-frozen in liquid ethane using a ThermoFisher Scientific Vitrobot Mark IV set to 90% humidity at 10 °C (blotting force −5, blotting time 3 to 3.5 s). Movies were recorded in underfocus on a 200 kV Talos Arctica G2 (ThermoFisher Scientific) electron microscope equipped with a Bioquantum K3 (Gatan) detector operated by EPU (ThermoFisher Scientific).

### Single-Particle Image Processing and Helical Reconstruction.

Movie frames were gain corrected, dose weighted, and aligned using cryoSPARC Live (75). Initial 2D classes were produced using the auto-picker implemented in cryoSPARC Live. The following image processing steps were performed using cryoSPARC. The best-looking classes were used as templates for the filament trace. The resulting filament segments were extracted with 600 px box size (Fourier cropped to 200 px) and subjected to multiple rounds of 2D classification. The remaining segments were reextracted with a box size of 400 px (Fourier cropped to 200 px) and subjected to an additional round of 2D classification. The resulting 2D class averages were used to determine filament diameters and initial symmetry guesses in PyHI (76). Symmetry guesses were validated by initial helical refinement in cryoSPARC and selection of the helical symmetry parameters yielding reconstructions with typical PspA features and the best resolution. Then all segments were classified by heterogeneous refinement and subsequent 3D classifications using the initial helical reconstructions as templates. The resulting class distribution gave the PspA rod diameter distribution shown in Fig. 2. The resulting helical reconstructions were subjected to multiple rounds of helical refinement including the symmetry search option. For the final polishing, the segments were reextracted at 400 px without Fourier cropping. Bad segments were discarded by heterogeneous refinement. Higher-order aberrations were corrected by global and local CTF refinement followed by a final helical refinement step. The local resolution distribution and local filtering was performed using cryoSPARC (*SI Appendix*, Fig. S3 *A*–*C*). The resolution of the final reconstructions was determined by Fourier shell correlation (auto-masked, FSC = 0.143) (*SI Appendix*, Fig. S3*B*).

### Cryo-EM Map Interpretation and Model Building.

The 3D reconstructions were B-factor sharpened in Phenix (*phenix.auto-sharpen*) (77). The handedness of the final map was determined by rigid-body fitting the PspA reference structure aa 22 to 217 (PDB:7ABK) (11) into the final maps using ChimeraX (78, 79) and flipped accordingly. 7ABK was flexibly MDFF fitted to the 3D reconstructions using ISOLDE (80). Some of the structures in the presence of EPL showed additional density at the tip of α1 which we interpreted as the additional N-terminal residues. Therefore, helix α0 (aa 1 to 22) was built from scratch, joined with α1 and flexibly MDFF fitted to the 3D reconstructions with helix restraints from aa 2 to 9 and aa 11 to 21 using ISOLDE (80). Then, the respective helical symmetry was applied to all models to create assemblies of 60 monomers each. The assembly models were subjected to auto-refinement with *phenix.real_space_refine* (81) (with NCS constraints and NCS refinement). After auto-refinement, the new models were used for local model-based map sharpening with LocSCALE (82) to produce the final maps. The auto-refined models were checked/adjusted manually in Coot (83) and ISOLDE (80) before a final cycle of auto-refinement with *phenix.real_space_refine* (81) (with NCS constraints and NCS refinement). After the final inspection, the model was validated in *phenix.validation_cryoem* (84)*/Molprobity* (85). Image processing, helical reconstruction, and model building were completed using SBGrid-supported applications (86). High-performance computing was performed at the supercomputer JURECA of Forschungszentrum Jülich (87). In this manner, a total of 15 cryo-EM maps were determined in 2 samples (Table S1 and S3).

## Supplementary Material

Appendix 01 (PDF)

## Data Availability

The EMDB accession numbers for cryo-EM maps and PspA models are EMD IDs: 52526 (88), 52527 (89), 52528 (90), 52529 (91), 52530 (92), 52531 (93), 52532 (94), 52533 (95), 52534 (96), 52535 (97), 52536 (98), 52537 (99), 52538 (100), 52539 (101), 52540 (102), and 52541 (103), and PDB-IDs: 9HZM (88), 9HZN (89), 9HZO (90), 9HZP (91), 9HZQ (92), 9HZR (93), 9HZS (94), 9HZT (95), 9HZU (96), 9HZV (97), 9HZW (98), 9HZX (99), 9HZY (100), 9HZZ (101), 9I00 (102), and 9I01 (103). All study data are included in the article and/or *SI Appendix*.
