# Supplementary material for: The bacterial ESCRT-III PspA rods thin lipid tubules and increase membrane curvature through helix α0 interactions
Source: Proc Natl Acad Sci U S A. 2025 Aug 4;122(32):e2506286122. doi: 10.1073/pnas.2506286122 (PMC12358876; doi:10.1073/pnas.2506286122)
Supplement: Supplementary file 1 — Appendix 01 (PDF) [file pnas.2506286122.sapp.pdf]

Supporting Information for

The bacterial ESCRT-III PspA rods thin lipid tubules and increase membrane curvature through helix α0 interactions

Esther Hudina[#1], Stephan Schott-Verdugo[#], Benedikt Junglas[#], Mirka Kutzner, Ilona Ritter, Nadja Hellmann, Dirk Schneider, Holger Gohlke[*], Carsten Sachse[*]

[#]These authors contributed equally
* Correspondence: c.sachse@fz-juelich.de (C.S.)
gohlke@uni-duesseldorf.de (H.G.)

Supporting Text 2
Biophysical characterization of PspA-helix α0 interactions with membranes 2
Supporting Material and Methods 2
Membrane binding of the peptides analyzed by CD and Trp fluorescence spectroscopy 2
Adaptive steered and umbrella sampling MD simulations 3
Free energy estimation from potential of mean force 4
Backmapping of CG 290 Å diameter PspA / membrane structures and all-atom MD simulations 6
Membrane curvature analysis 7
Cryo-electron tomography and tomogram reconstructions 8
Analysis and visualization tools 8
Supporting Tables 10
Table S1. Data collection, image processing and model refinement PspA EPL 10
Table S2. Layer line properties of SynPspA + EPL rods 11
Table S3. Data collection, image processing and model refinement PspA α1-5 EPL 11
Table S4. Sample details of cryo-EM specimens 13
Supporting Figures 14
Figure S1: Interaction of PspA's isolated helix α0 with membranes. 14
Figure S2: Helix α0 metrics in unbiased molecular dynamics (MD) and umbrella sampling (US) simulations 15
Figure S3: Local Resolution maps and FSC curves of cryo-EM PspA structures. 16
Figure S4: Helical lattice and power spectra of PspA + EPL structures. 17
Figure S5: PspA rod density, radial density profiles and mass per length. 18
Figure S6: The 290 Å PspA bilayer complex modeled in the full-atom representation. 19
Figure S7: Helfrich bending energy for 2D Gaussians of different amplitudes and standard deviations 20
Figure S8: Tomograms of PspA with EPL membranes. 21
References 21

Supporting Text

Biophysical characterization of PspA-helix α0 interactions with membranes

To date, the mechanism of PspA interaction with membranes has not been fully characterized as the full-length PspA cryo-EM structure did not contain density for helix α0 (1). Previous studies suggested that the isolated N-terminal peptide α0 adopts an α-helix structure in the presence of negatively charged lipids (2, 3). To understand the role of helix α0 in membrane interaction and to determine the amino acids relevant for binding to negatively charged lipids, we tested the isolated PspA helix α0 peptide (residues 1-22) and a R6A and R9A (PspA α0 RRAA) mutant with replaced positively charged residues and analyzed changes in secondary structure using circular dichroism (CD) spectroscopy (**Suppl. Fig. 1A**). Upon increasing the EPL concentration, wild-type (WT) α0 revealed a significant reduction in the signal intensity at 220 nm showing formation of α-helical structure induced by membrane-adhesion of the peptide (**Suppl. Fig. 1B**). Based on the CD data, the α-helix content of the PspA WT α0 peptide was estimated to be ~52 % at high EPL concentrations, while it was barely present (3.2 %) in the PspA-α0-RRAA sample. The signal intensity at 220 nm did not change in the case of the PspA-α0-RRAA peptide when membranes were added, indicating that its secondary structure is not changing, *i.e.* the petide does not bind to membranes.

To investigate whether PspA-α0-RRAA can still bind to membrane surfaces, we additionally monitored changes in the tryptophan (Trp) fluorescence in the presence of EPL membranes. The α0 peptide contains a single Trp residue located in the hydrophobic face of the α-helix projection (see **Suppl. Fig. 1A**), which likely inserts into the membrane upon helix α0 membrane adhesion. Notably, only the WT, but not the PspA-α0-RRAA peptide, displayed a shift in the Trp fluorescence signal when EPL membranes were added, again indicating that solely the WT-α0-PspA peptide interacts with EPL membranes, whereas the α0 RRAA mutant did not (**Suppl. Fig. 1C**). Based on the binding curves, the free energy of binding of the WT peptide was estimated to be between ΔG^0 = -3.9 and -3.7 kcal mol^{-1} and between ΔG^0 = -5.7 and -5.3 kcal mol^{-1} for CD spectroscopy and Trp fluorescence, respectively. In conclusion, our results show that the isolated PspA-α0 peptide binds to membrane surfaces, which involves the concomitant formation of an α-helical structure. The positively charged residues R6 and R9 are critical for helix α0 binding to negatively charged membrane surfaces.

Supporting Material and Methods

Membrane binding of the peptides analyzed by CD and Trp fluorescence spectroscopy

The peptides PspA α0 WT [MELFNRVGRVLKSQLTHWQQQQEA] and PspA α0 RRAA [MELFNAVGAVLKSQLTHWQQQQEA] were purchased from PSL GmbH (Heidelberg, GER). This sequence corresponds to the first 24 amino acids of the *Synechocystis* PspA protein. Membrane binding

of the peptides was investigated via monitoring Trp fluorescence changes. 2 µM peptide (10 mM Tris, pH 8.0) was incubated with increasing amounts of liposomes up to 200 µM lipid. The liposomes were prepared as described above. Upon incubation at 25°C for 15 min, corrected Trp fluorescence emission spectra were recorded using a fluorescence spectrometer (FP-8500, Jasco, Pfungstadt, Germany), from 300 nm to 450 nm with an excitation wavelength of 280 nm and an increment of 0.2 nm. The excitation and the emission slit widths were set to 2.5 nm. Buffer spectra were subtracted. The influence of lipid binding on the secondary structure of the peptides was determined via circular dichroism (CD) spectroscopy using a J-1500 CD-spectrometer (Jasco, Pfungstadt, Germany). 34 µM peptide (10 mM Tris, pH 8.0) was incubated with increasing amounts of liposomes, with lipid concentrations set to yield a similar lipid/peptide ratio as in the fluorescence spectroscopy measurements. After incubation at room temperature for 15 min, spectra were recorded in 1 nm steps at 25°C. Three individual samples were measured for each sample; 12 spectra were averaged. The secondary structure content of the peptides was calculated based on the CD spectroscopy data using BeStSel (4).

Peptide/membrane binding was quantitatively analyzed by fitting a hyperbolic binding model to the data, expressing the fraction of bound peptide as a function of lipid/peptide ratio r:

$$S = S_o + \Delta S \frac{r}{r + K}$$

with S either reflecting fluorescence intensity or ellipticity. Thus, K is related to the respective K_D value via $K_D = K\, c_o$, with c_o reflecting the peptide concentration in the given experiment (2 µM and 34 µM, respectively). The binding free energy calculations are based on the K_D values, with $\Delta G^o = RT*\ln(K_D)$, with K_D in M^{-1}.

Adaptive steered and umbrella sampling MD simulations

In all replicas, the peptide bound quickly to the membrane surface (see main text). From the last frame of the first replica, adaptive steered molecular dynamics (AsMD) simulations were started, using the distance along the z-axis between the center of mass (COM) of the C_α atoms of the peptide and the COM of the phosphorous atom of the phospholipid bilayer as the reaction coordinate (RC) with a force constant of 100 kcal mol^{-1} Å^2. Twelve iterations of pulling the peptide were performed, increasing the RC distance by 2.5 Å over 10 ns in each (0.25 Å ns^{-1}), from 20.3 to 50.3 Å. In each iteration, 40 replicas were performed in parallel, selecting the one with a work profile closest to the Jarzynski average, as previously described (5, 6) his process allows to obtain a trajectory of a low free energy from the bound to the unbound state. To further obtain a free energy profile by means of umbrella sampling (US), intermediate structures were extracted every 1 Å and another window closer to the membrane center was added, resulting in 32 windows at a z-axis distance between 19.3 and 50.3 Å. The peptide was restrained with a harmonic potential at the corresponding RC value of a window using a force constant of 5 kcal mol^{-1} Å^2. 1 µs of US was used per window, recording the RC every 4 ps. Samples showed a

low correlation, as the highest autocorrelation time was 40 ps, as tested for window 3. The free energy profile or potential of mean force (PMF) was calculated by using the weighted histogram analysis method as implemented in the WHAM program version 2.0.11 (7). The final PMF and its error were obtained by taking the samples of the last 500 ns, averaging and calculating the standard deviation by generating 50 independent PMF calculations with 10 ns chunks and setting the value at the water bulk to zero.

Free energy estimation from potential of mean force

For calculating the free energy of binding, the adsorption coefficient κ to the membrane surface was described by a modified approximation taken from Ben-Tal et al., 2000.

$$\kappa = \frac{\frac{1}{\lambda}\int_o^\lambda dz \int e^{-\beta W(z,Y)}\, dY}{8\pi^2} \approx \frac{\Omega\frac{1}{\lambda}\int_o^\lambda e^{-\beta w(z)} dz}{8\pi^2} \qquad \text{eq. 1}$$

where W is the binding potential, which depends on the distance to the membrane (z) and the orientation of the peptide as described by Euler angles (Y, with pitch, roll and yaw being θ, φ and ψ, respectively) and λ is the membrane distance that defines a bound state. To calculate the pitch and roll angles, two reference vectors were constructed: a vector from the peptide's center of mass to the center of mass of the first residue (v_1) and one from the center of mass between the C_α atoms of residues 2 to 5 to the C_α atom of residue 2 (v_2). The pitch was calculated as the angle between v_1 and the membrane normal, while the roll was calculated by aligning v_1 onto $\hat{x}$ and calculating atan2($v_{2\hat{z}}$,$v_{2\hat{y}}$), where $v_{2\hat{z}}$, $v_{2\hat{y}}$ are x and y components of v_2. By binding to the membrane surface, three of the six global degrees of freedom of the peptide are constrained, the position along the membrane normal and the pitch and the roll with respect to the membrane normal (**Suppl. Fig. 2C and D**). The calculated PMF $w(z)$ captures the free energy as a function of the distance to the membrane, but not the dependence on Y. To consider the constraint in the pitch and roll, the bound state range was considered by Ω on the right side of eq. 1

$$\Omega = 2\pi \frac{\sum_i^N[\cos(\theta_i)_{2.5} - \cos(\theta_i)_{97.5}]}{N} \frac{\sum_i^N[\varphi_{i_{97.5}} - \varphi_{i_{2.5}}]}{N} \qquad \text{eq. 2}$$

where 2π represents that any yaw angle is possible, the center term is the average span of pitch angles and the right term is the average span of the roll angles sampled on the windows in the bound state with N states, with the values 2.5 and 97.5 depicting the corresponding percentile values. Similar approaches have been used to estimate the impact of binding on rotational entropy (9). As almost 100% of the integral of $\int_o^\lambda e^{-\beta w(z)} dz$ is recovered when using windows that consider a RC $\leq$ 25 Å (**Suppl. Fig. 2G**),

the first eight windows with RC values restraint between 19.3 and 27.3 Å were used, resulting in Ω = 2.19.

From κ, the binding free energy is obtained (8)

$$\Delta G = -RTln\left(\kappa\left(\frac{V_b}{V_f}\right)\right) \qquad \text{eq.3}$$

where V_b and V_f correspond to the bound and free volumes, respectively. Considering the standard state volume $V_f = V_o$ = 1661 Å^3, which corresponds to a concentration of 1 M, that the interaction with the membrane only constrains one translational dimension (10) and that the bound distance along the membrane normal is λ, *i.e.*, $V_b = \lambda A$, with A being the membrane surface area,

$$\Delta G^o = -RTln\left(\kappa\left(\frac{\lambda}{\sqrt[3]{V_o}}\right)\right) \approx -RTln\left(\frac{\Omega\int_o^\lambda e^{-\beta w(z)}dz}{\sqrt[3]{V_o}\,8\pi^2}\right) \qquad \text{eq.4}$$

Eq. 4 results in ΔG^0 = -18.55±2.07 kcal mol^{-1} for T = 300 K, which is in a similar order of magnitude as the distance term of a recent thorough multiple collective variable calculation for Phospholipase C, a peripheral membrane protein, but not as the overall binding free energy (11). This is because the approximation reflects the binding of a rigid peptide, considered a rigid cylinder, to the membrane surface (as explained in (12)), but does not take into account the contribution due to conformational changes that happen to the peptide upon binding. In this case, the amphipathicity of helix α0 leads to a helix formation upon binding, which is entropically disfavorable, as the number of available conformational states is reduced. Furthermore, this effect is not captured by the distance reaction coordinate. At the plateau in the PMF, the sudden increase in reachable conformations does not cause a decrease in the free energy, as it becomes independent of the distance to the membrane surface (**Fig. 2D and Suppl. Fig. 2B-D)**. To estimate this contribution, the *k* nearest neighbors approach of the method PDB2ENTROPY (13) was used to estimate the entropy of residue torsions. For the analysis, we considered as bound structures windows 1 to 25, i.e., with restrained distances to the membrane center between 19.3 and 43.3 Å, and unbound structures those of windows 26 to 32, i.e, restrained between 44.3 and 50.3 Å. The rationale for this selection was that structures in windows 26 to 32 represent those mostly at the plateau of the PMF profile (**Fig. 1E**) and show a clear detachment along the trajectories, as evidenced by the increase in RMSD with respect to the bound structure and change in Euler angles (**Suppl. Fig. 2B-D**). This results in $T\Delta S_{\text{unbinding,conf.}}$ = 12.23 ± 0.04 kcal mol^{-1} for T = 300 K (**Suppl. Fig. 2F**). Together with the result of eq. 4, the standard binding free energy of helix α0 is estimated as $\Delta G^0_{\text{binding}}$ = -6.32±2.07 kcal mol^{-1}.

Backmapping of CG 290 Å diameter PspA / membrane structures and all-atom MD simulations

Two intermediate structures of the 290 Å complex with the membrane pulled were selected for all-atom simulations, one with the membrane 'half-way-through' and another with the membrane 'all-way-through' (HWT and AWT, see main text). To simulate at the all-atom level, the coordinates have to be properly backmapped from the CG level. For the protein, the VMD SIRAH tools plugin was used (14). For the lipids, on the other hand, no automated solution is available for SIRAH lipids, partially, because the positions of the full-atom representation are ill-defined by the CG beads. To accomplish the backmapping, the following algorithm was implemented:

1) Coordinates of each lipid were extracted independently as PDB files.
2) Each bead was renamed according to the AMBER Lipid21 equivalent, as defined in the amber_lipid.map file included in AmberTools 23. PS residues were backmapped to PG residues, to recover a proper Gram-negative membrane.
3) Each lipid was parametrized with LEaP, letting the program add missing atoms according to the force field definition.
4) Each lipid was minimized independently with pmemd for a maximum of 5000 steps, with 2500 steps of steepest descent minimization and using igb = 1. All coordinates that were directly backmapped from the CG structure were restrained with a positional restraint of 5 kcal mol^{-1} $Å^2$. To ensure proper structural isomers and proper stereochemistry, additional NMR restraints were imposed on acyl chain double bonds and the glycerol moiety of the head group.
5) The backmapped protein, all minimized lipids and ions were concatenated to reconstruct a backmapped system only lacking water.
6) The system was reparametrized with LEaP, imposing the box dimensions from the original CG simulation box.
7) A minimization of the concatenated system was performed with pmemd.MPI, imposing positional restraints on the previously restrained lipid atoms, ions and N/O/C_α backbone atoms of the protein, for 2500 steps of steepest descent and conjugated gradient minimization, respectively.
8) The minimized structure was resolvated using Packmol (15), adding 11 water molecules for each WT4 residue in the original CG system, as expected for the SIRAH water model (16). A box smaller by 0.5 Å in each dimension was used, to avoid potential overlaps with periodic images.

The resulting structures were reparametrized with LEaP and simulated using the same simulation parameters as for helix α0, totaling over 5,380,000 atoms. Due to the system size, the minimization protocol used for helix α0 was not practical. At the same time, as the protein and membrane structures come mostly from stable CG simulations and previous minimization runs, mostly water clashes had to

be removed. For this, a single pmemd.cuda minimization run with SHAKE enabled was performed, allowing a maximum of 10,000 steepest descent/conjugated gradient steps. Further thermalization and simulation conditions were the same as those used for helix α0 (see above). Eight production replicas of 300 ns length were performed.

Membrane curvature analysis

Membrane bilayers display elastic characteristics, which resist mechanical bending and stretching. While stretching motions are harder to accomplish, bending motions occur due to thermal fluctuations and/or interactions, e.g., with binding partners. The energetic cost E of bending a membrane patch is described by the Helfrich Hamiltonian (17, 18).

$$E = \int dA \left[\frac{1}{2}\kappa(K - K_0)^2 + \bar{\kappa} K_G\right] \qquad K = c_1 + c_2 \qquad K_G = c_1 c_2 \qquad \text{eqs. 5,6,7}$$

where c_1 and c_2 are the main local curvatures, K is the total curvature (K / 2 is the mean curvature H), K_G is the Gauss curvature, κ is the monolayer bending modulus and $\bar{\kappa}$ is the monolayer Gauss curvature modulus. K_0 corresponds to the spontaneous curvature, which is 0 for symmetric bilayers. The integral of the Gauss curvature is 0 for a smooth periodic plane due to symmetry and is a constant for compact surfaces (18). Both moduli are quantities difficult to obtain (19) and to our knowledge, not available for a membrane with a composition DOPE:DOPG 3:1. Here, a value of 12 kT was used for κ, as determined by X-ray diffraction for DOPE monolayers (20). It has been shown from simulations that $-\bar{\kappa} / \kappa$ for DOPE is ~0.92 (18) and we took $\bar{\kappa} = -\kappa$ for simplicity.

Points representing the surface of each leaflet were obtained using Memsurfer (21), which triangulates surface points (phosphorous atoms) to generate a surface mesh and uses The Visualization Toolkit (VTK) to obtain their curvatures (22). Due to fluctuations of lipids on the surface and local high curvatures, the surfaces obtained with default options generated regions with curvature artifacts, particularly in the lower leaflet. To tackle this, the scripts were further modified to add two additional smoothing filters of the retrieved surface points using the VTK python interface: a Laplacian smoothing filter (vtkSmoothPolyDataFilter) and a constrained smoothing filter (vtkConstrainedSmoothingFilter), setting as a constraint distance z-score / 10 of the mean curvature at any given point. While the so generated surfaces are smooth, they include local fluctuations characteristic of Brownian motion, which adds noise when calculating the bending energy (eq. 5).

To measure the curvature caused by the protein and remove noise, the surfaces were fitted to a 2D Gaussian (eq. 8):

$$f(x, y) = A e^{\frac{-(x-x_0)^2}{2\sigma_x^2}} e^{\frac{-(y-y_0)^2}{2\sigma_y^2}} + S \qquad \text{eq. 8}$$

where x_0 and y_0 are the center and σ_x and σ_y the variances or spread on the corresponding axis directions, A is the amplitude and S is a shift term. By expressing $f(x,y)$ implicitly as $F(x,y,z) = 0$, the mean curvature per unit area was calculated (23) (eq. 9)

$$H(x,y,z) = -\frac{1}{2}\nabla \cdot \left(\frac{\nabla F)}{|\nabla F|}\right) \qquad \text{eq. 9}$$

which is the divergence of the normal unit vector to the surface defined by F. The Gauss curvature is calculated from the following expression (24)

$$K_G(x,y,z) = \{[F_z(F_{xx}F_z - 2F_xF_{xz}) + F_x^2F_{zz}][F_z(F_{yy}F_z - 2F_yF_{yz}) + F_y^2F_{zz}] - [F_z(-F_xF_{yz} + F_{xy}F_z - F_{xz}F_y) + F_xF_yF_{zz}]^2\}[F_z^2(F_x^2 + F_y^2 + F_z^2)^2]^{-1} \qquad \text{eq. 10}$$

where the subscript denotes a partial derivative. The Gauss curvature term was only calculated for partial surface integrals, as the total for periodic surfaces is 0, as already mentioned (**Fig. 4E**; note the plateau to 0 at higher integration areas).

Cryo-electron tomography and tomogram reconstructions

Tilt series for cryo-electron tomography were recorded on a 300 kV Titan Krios G4 (ThermoFisher Scientific) electron microscope equipped with a Biocontinuum K3 (Gatan) detector operated by Tomo (ThermoFisher Scientific) using a dose-symmetric scheme at -60° to 60° with 3° steps. Tilt images were acquired at a magnification of 64 kx (pixel size 1.362 Å) with a nominal underfocus of 4.0 µm. The total dose for each tilt series was 131 $e^-/Å^2$. A total of 20 tilt series were collected. Tilt image frames were gain-corrected, dose-weighted and aligned using WARP (25). The resulting tilt series were aligned, 8x binned and reconstructed by the weighted back projection method using AreTomo (26). The tomograms were segmented in Dragonfly (Object Research Systems) by progressively training a U-Net with an increasing number of manually segmented tomogram frames (5-15). Then the trained U-Net was used to predict those features of the tomogram. For visualization, the segmentation was cleaned up by removing isolated voxels of each label group (islands of <150 unconnected voxels). The resulting segmentation was imported to ChimeraX (27) for 3D rendering.

Analysis and visualization tools

Diameters of the final PspA rod reconstructions were measured using ImageJ (28): First radial intensity profiles of each reconstruction were created (**Suppl. Fig. 3A**). Then the radius at an intensity cutoff of 0.3 was read. The radius readout from reconstructions from the same helical symmetry class was

averaged, converted to diameters and rounded to 5 Å increments. Outer and inner leaflet radii of engulfed membrane tubes were determined from the same radial intensity profiles at the peak maxima from each bilayer leaflet. Metrics obtained from molecular simulations and trajectories were handled with CPPTRAJ (29). Visualization of structures was done using Open-Source PyMOL version 2.4 (DeLano, 2002), ChimeraX 1.6.1 (27) and VMD 1.9.4 VM (32). The fitting process and all calculations were done using Python 3 and NumPy (33), SciPy (34) and SymPy (35). Data and statistical analysis were performed using OriginPro 2021b (OriginLab Corp., Northampton, USA). Detailed descriptions of quantifications and statistical analyses (exact values of *n*, dispersion and precision measures used, and statistical tests used) can be found in the respective Figures, Figure legends and Methods section.

Supporting Tables

Table S1. Data collection, image processing and model refinement PspA EPL

	200 Å	215 Å	235 Å	250 Å	270 Å	280 Å	290 Å	305 Å	320 Å	345 Å
Movies	3,674									
Magnification	63kx									
Voltage (kV)	200									
Total dose (e^{-}/ Å ^{2})	58									
Defocus range (μm)	1.5 – 3.5									
Physical pixel size (Å)	1.362									
Detector	Gatan K3									
Symmetry imposed	C2 5.78 Å 34.4 º	C1 2.56 Å 130.2 º	C3 7.01 Å 28.2 º	C1 2.12 Å -83.6 º	C2 3.94 Å -78.0 º	C3 5.61 Å 22.9 º	C4 6.85 Å 21.2 º	C1 1.61 Å -85.0 º	C1 1.53 Å -140.3 º	C1 1.46 Å 75.5 º
Final no. of segments (ASUs)	45,586 (455,860)	27,620 (635,260)	52,698 (474,282)	34,131 (819,144)	57,924 (868,860)	112,414 (1,236,554)	113,665 (1,022,985)	72,098 (2,667,626)	69,652 (2,716,428)	31,073 (279,657)
Global map resolution (Å, FSC = 0.143)	4.7	5.8	6.2	6.1	5.5	5.3	4.7	4.6	5.2	6.5
Local map resolution range (Å, FSC = 0.5)	5.6 - 8.5	6.2 - 8.6	6.3 - 8.8	6.4 - 7.8	6.2 - 10.8	5.7 - 9.1	5.3 - 8.7	5.4 - 8.6	6.0 - 9.4	6.9 - 11.2
Initial model used (PDB code)	7ABK									
**Model refinement**										
Model resolution (Å)	4.3	5.4	6.1	6.2	6.3	5.5	4.6	4.6	5.3	7.1
CC mask	0.81	0.81	0.76	0.80	0.85	0.82	0.81	0.81	0.82	0.73
CC box	0.89	0.85	0.80	0.84	0.88	0.85	0.86	0.85	0.84	0.59
CC peaks	0.77	0.73	0.63	0.67	0.71	0.68	0.71	0.67	0.66	0.21
CC volume	0.81	0.82	0.75	0.80	0.84	0.82	0.82	0.81	0.82	0.74
CC ligands	-	-	-	-	-	-	-	-	-	-
Map sharpening B-factor (Å^{2})	190.65	203.29	258.86	163.99	187.03	275.49	306.71	211.32	248.51	258.90
**Model composition**										
Nonhydrogen atoms	94,680	94,680	94,680	94,680	94,680	105,600	105,600	105,600	105,600	105,600
Protein residues	11,760	11,760	11,760	11,760	11,760	13,020	13,020	13,020	13,020	13,020
**RMSDs**										
Bond lengths (Å)	0.003	0.001	0.003	0.002	0.002	0.002	0.001	0.002	0.002	0.003
Bond angles (°)	0.400	0.350	0.489	0.367	0.421	0.487	0.344	0.439	0.406	0.636
**Validation**										

MolProbity score	1.47	1.20	1.59	1.45	1.65	1.65	1.42	1.47	1.58	1.25
Clashscore	8.64	4.20	12.07	8.28	13.99	13.98	7.68	8.67	8.06	4.75
Rotamer outliers (%)	0.00	0.61	0.00	0.00	0.00	0.00	0.00	0.00	0.00	0.00
**Ramachandran plot**										
Favored (%)	98.97	98.45	98.45	100.00	98.45	98.14	98.60	98.14	97.21	98.60
Allowed (%)	1.03	1.55	1.55	0.00	1.55	1.86	1.40	1.86	2.79	1.40
Disallowed (%)	0.00	0.00	0.00	0.00	0.00	0.00	0.00	0.00	0.00	0.00
**Deposition IDs**										
EMDB	52532	52533	52534	52535	52536	52537	52538	52539	52540	52541
PDB	9HZS	9HZT	9HZU	9HZV	9HZW	9HZX	9HZY	9HZZ	9I00	9I01

Table S2. Layer line properties of SynPspA + EPL rods

	200 Å	215 Å	235 Å	250 Å	270 Å	280 Å	290 Å	305 Å	320 Å	345 Å
**Bessel order 1.**	10	11	12	13	14	15	16	17	18	19
Layer Line position (1/ Å)	121.1	92.3	118.4	109.0	121.1	115.9	115.9	113.5	113.5	113.5
**Bessel order 2.**	2	3	3	4	4	3	4	4	5	5
Layer Line position (1/ Å)	30.3	30.8	29.6	30.3	30.3	29.0	29.0	28.4	28.4	28.4

Table S3. Data collection, image processing and model refinement PspA α1-5 EPL

	235 Å	250 Å	270 Å C1	270 Å C2	280 Å	290 Å
Movies	3,149					
Magnification	49kx					
Voltage (kV)	200					
Total dose (e^{-}/ Å ^{2})	44					
Defocus range (μm)	1.0 – 3.5					
Physical pixel size (Å)	0.868					
Detector	Gatan K3					
Symmetry imposed	C4 9.03 Å 27.6 º	C1 2.10 Å -83.6 º	C1 1.92 Å 76.7 º	C2 3.93 Å -78.0 º	C5 9.03 Å 21.9 º	C4 6.85 A 21.1 º
Final no. of segments (ASUs)	121,749 (852,243)	1,405,435 (19,676,090)	629,133 (10,066,128	568,968 (4,551,744)	636,672 (1,910,016)	166,757 (1,500,813)
Global map resolution (Å, FSC = 0.143)	4.2	3.8	3.9	3.9	3.9	5.4

Local map resolution range (Å, FSC = 0.5)	4.4 - 5.1	3.5 - 4.8	3.9 - 5.1	3.9 - 5.2	4.0 - 5.2	5.5 - 6.4
Initial model used (PDB code)	7ABK					
**Model refinement**						
Model resolution (Å)	4.1	3.7	3.8	3.9	3.8	5.0
CC mask	0.81	0.77	0.74	0.76	0.79	0.75
CC box	0.88	0.84	0.82	0.83	0.86	0.83
CC peaks	0.77	0.70	0.670	0.72	0.74	0.66
CC volume	0.81	0.76	0.74	0.76	0.79	0.76
Map sharpening B-factor (Å^2)	147.92	29.07	97.75	115.88	128.22	240.89
**Model composition**						
Nonhydrogen atoms	93,600	93,600	93,600	93,600	93,600	93,600
Protein residues	11,640	11,640	11,640	11,640	11,640	11,640
**RMSDs**						
Bond lengths (Å)	0.002	0.002	0.003	0.004	0.002	0.003
Bond angles (°)	0.374	0.480	0.643	0.881	0.436	0.477
**Validation**						
MolProbity score	1.44	1.54	1.53	1.36	1.40	1.53
Clashscore	8.03	10.42	10.21	6.50	6.97	10.11
Rotamer outliers (%)	0.00	0.31	0.00	0.75	0.62	0.62
**Ramachandran plot**						
Favored (%)	98.96	98.30	98.44	100.00	97.92	98.44
Allowed (%)	1.04	1.70	1.56	0.00	2.08	1.56
Disallowed (%)	0.00	0.00	0.00	0.00	0.00	0.00
**Deposition IDs**						
EMDB	52526	52527	52528	52529	52530	52531
PDB	9HZM	9HZN	9HZO	9HZP	9HZQ	9HZR

Table S4. Sample details of cryo-EM specimens

	PspA apo	*PspA EPL*	*PspA* α*1-5 EPL*
Protein Conc.	6 – 8 mg/mL	1.2 mg/mL	8 mg/mL
Lipid Conc.	-	2.5 mg/mL	5.0 mg/mL
NTP Conc.	-	-	-
$MgCl_2$ Conc.	-	-	-
Magnification	63 kx	63 kx	49 kx
Pixel size	1.362 Å	1.362 Å	0.868 Å
Frames	40	40	70
Total dose	58 e−/Å2	58 e−/Å2	44 e−/Å2
Defocus range	1.0 to -3.0 µm	1.5 to 3.5 µm	1.0 to 3.5 µm
Movies	1,818	3,674	3,149

Supporting Figures

Supplement Figure 1

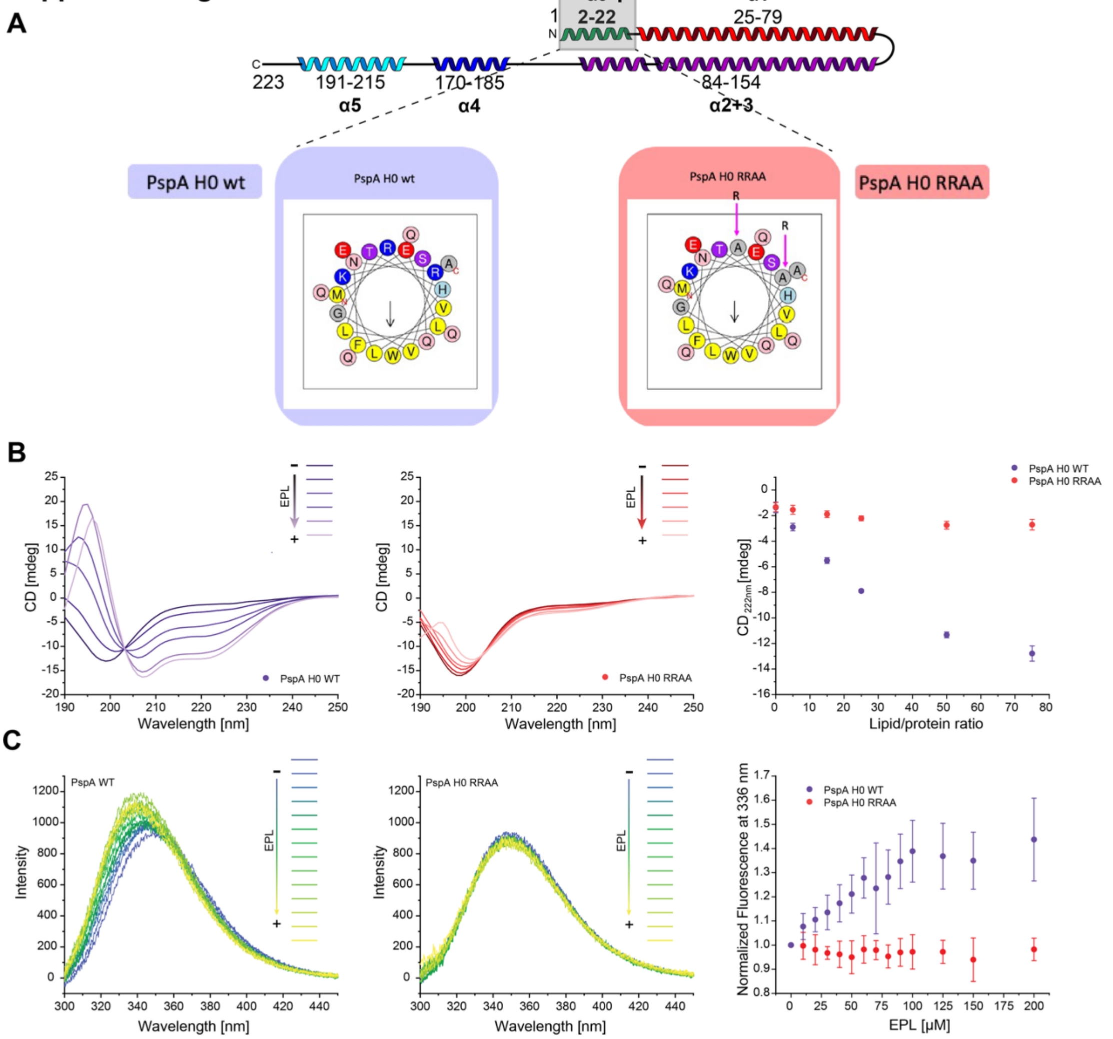

Figure S1: Interaction of PspA's isolated helix α0 with membranes.

A: Top: Secondary structure topology plot of the PspA ESCRT-III fold (α0 green with grey focus box, α1 red, α2+3 violet, α4 blue, α5 cyan). Bottom: Schiffer–Edmundson helix wheel projection of PspA (1-22) wild type (left) and PspA α0 RRAA (right), yellow hydrophobic residues, blue and red polar charged residues, light blue and red polar residues, grey neutral. **B**: Circular dichroism (CD) spectra of helix α0 in the presence of increasing EPL concentration (340 µM, 510 µM, 850 µM, 1700 µM and 2550 µM EPL), left: PspA wild-type (WT) in purple, center: PspA α0 RRAA mutant) in red indicate a membrane-induced increase in α-helix content in the WT depending on the lipid:protein ratio (right). The binding data obtained for the WT peptide by plotting the ellipticity at 222 nm versus the EPL/peptide ratio were analyzed based on a hyperbolic function yielding an apparent partition coefficient of 49.8 ± 8.2. **C**: Changes in the Trp fluorescence emission spectrum in the presence of EPL membranes. Left: PspA WT (purple), center: PspA α0 RRAA (red), right: fluorescence intensity at 336 nm as a function of lipid concentration. The binding data obtained by plotting the fluorescence intensities at 336 nm versus EPL/peptide ratio were analyzed based on a hyperbolic function, yielding an apparent partition coefficient of 47.0 ± 15.4. Thus, a lipid/peptide ratio similar to the value determined by CD spectroscopy is required to bind 50 % of the peptide. Based on these results, we estimated the free energy of binding of the WT peptide from the CD spectroscopy and Trp-based fluorescence analyses to be between ΔG^0 = -3.9 and -3.7 kcal mol^{-1}, and -5.7 and -5.3 kcal mol^{-1}, respectively.

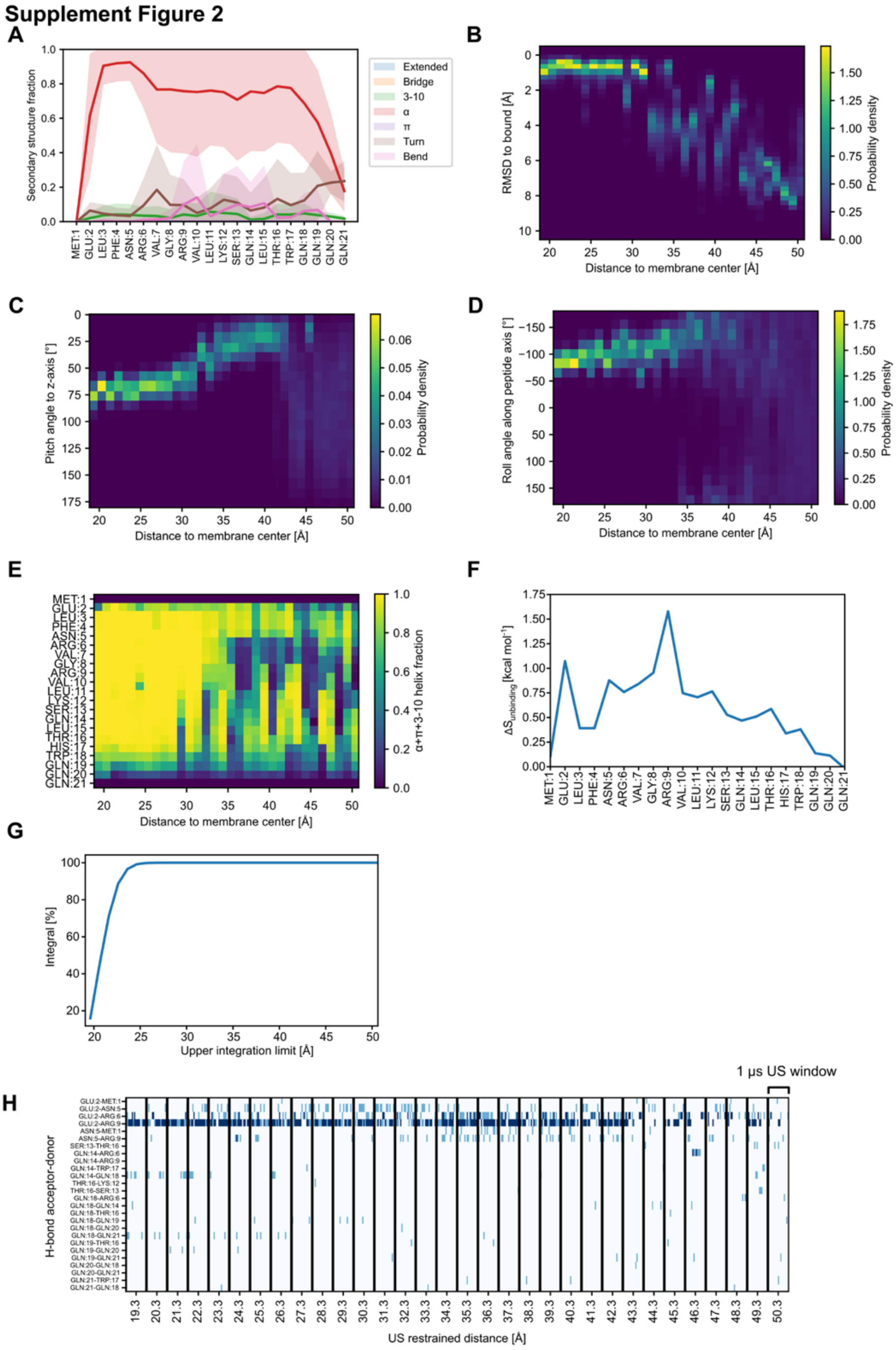

Figure S2: Helix α0 metrics in unbiased molecular dynamics (MD) and umbrella sampling (US) simulations.

A: Residue-wise mean secondary structure content over 12 replicas of unbiased MD simulations (as calculated by cpptraj) with the shaded area showing the standard deviation. On average, α0 forms an α-helix upon binding to the membrane surface. **B-E**: Analysis of peptide orientation and structural changes in the 32 US windows, with the reaction coordinate representing the distance to the membrane center along the z-axis (membrane normal). Along the reaction coordinate, the global degrees of freedom of helix α0 are restricted, as indicated by (**B**) the RMSD of the sampled structures, (**C**) the Euler angle describing the pitch with respect to the membrane normal and (**D**) the roll angle describing how much the peptide can roll around the peptide axis. **E**: As the peptide unbinds, the peptide transitions from a fully helical structure at the membrane surface to a partially unstructured conformation, with partial helical sections towards the N and C terminal ends. **F**: PDB2ENTROPY allows one to estimate conformational entropy changes between structure ensembles by performing a *k*-nearest neighbors analysis of the torsions of the peptide. **G**: The integration of the PMF (integral term in eq. 4) yields an almost constant value for an upper integration limit beyond 25 Å. **H**: Hydrogen bonds between side chains with a cumulative prevalence of at least 100 ns (10% of a single window). Light blue depicts a single H-bond, while dark blue shows that two H-bonds are involved in the interaction.

Supplement Figure 3

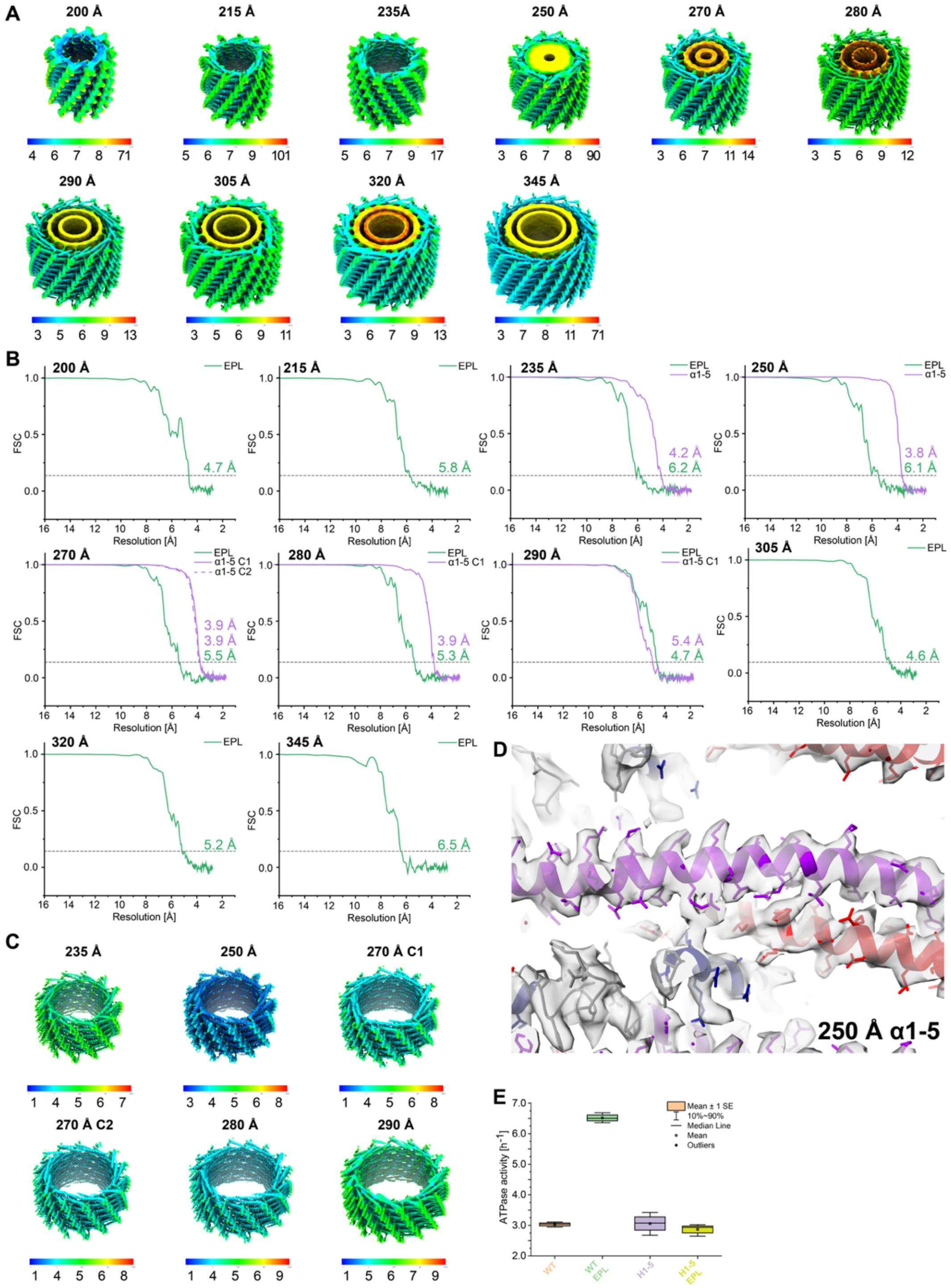

Figure S3: Local Resolution maps and FSC curves of cryo-EM PspA structures.

A: Local resolution maps of the helical PspA assemblies of the PspA + EPL sample with resolution displayed on the isosurface (scale indicated below). **B**: FSC curves of all PspA diameters of the data sets with PspA+EPL (green) and the data set with PspA α1-5 + EPL (violet) including the 0.143 resolution dashed threshold line. **C**: Local resolution maps of the helical PspA α1-5 + EPL assemblies with resolution displayed on isosurface (scale indicated below). **D**: 3.8 Å resolution density of 250 Å wide PspA α1-5 + EPL rod assembly with the refined atomic model of the monomer in ribbon representation (α1 red, α2+3 violet, α4 blue, α5 cyan). **E**: ATPase activity of the wild-type protein and the α1-5 mutant in the absence and presence of EPL; wild-type (WT) (orange): ATPase activity without EPL; WT EPL (green): ATPase activity in the presence of EPL; α1-5 variant (purple): ATPase activity of α1-5 variant in the absence of EPL; α1-5 EPL (yellow): ATPase activity of α1-5 variant in the absence of EPL; n=3.

Supplement Figure 4

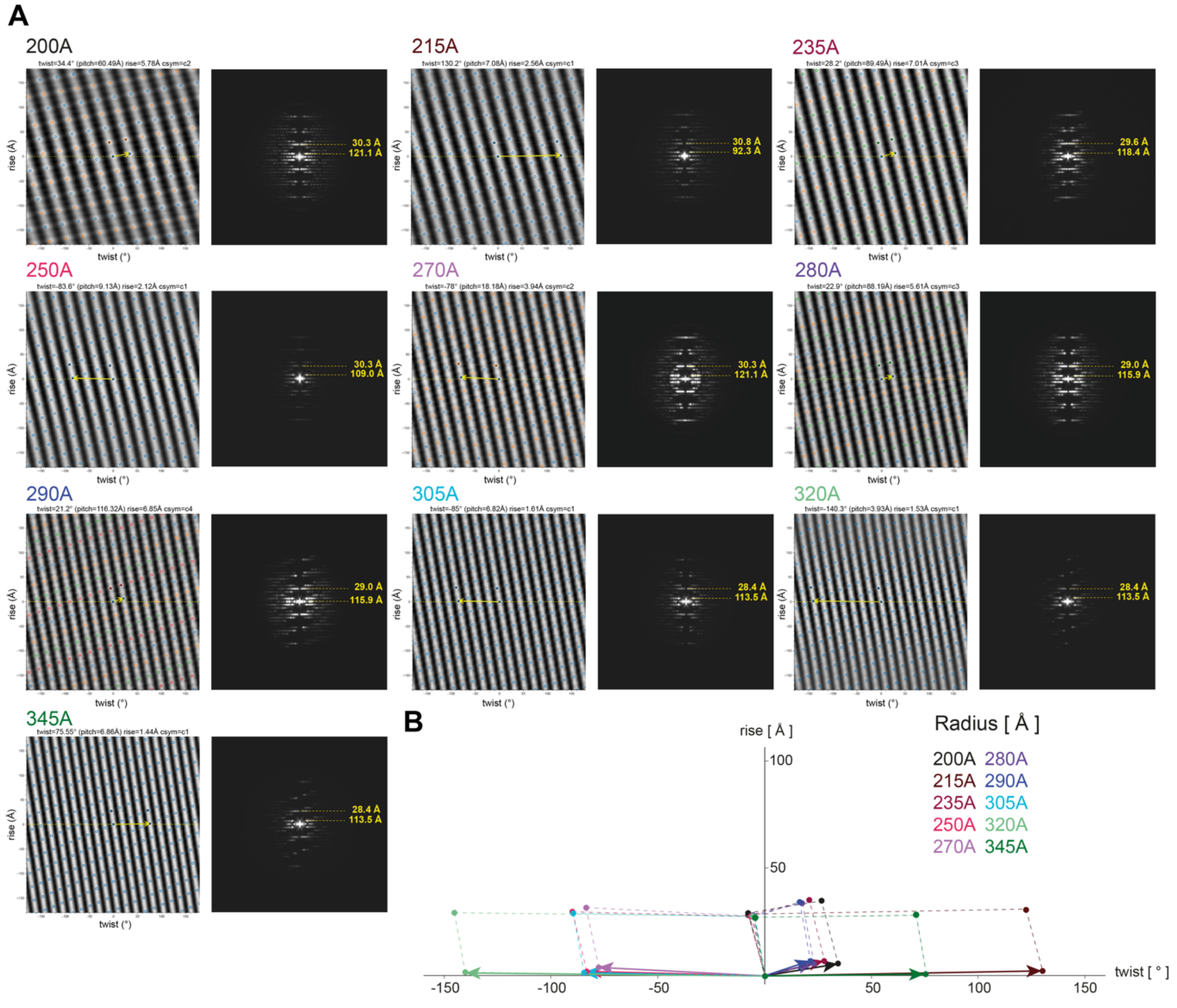

Figure S4: Helical lattice and power spectra of PspA + EPL structures.

A: Helix lattice plot (left) generated with HI3D (36), including unit cell of symmetry vectors and power spectra (right) generated using PyHI (37) together with used layer line position (yellow dashed lines) for PspA + EPL structure determination. **B**: Superimposed unit cells of the symmetry vectors of the different PspA diameters.

Supplement Figure 5

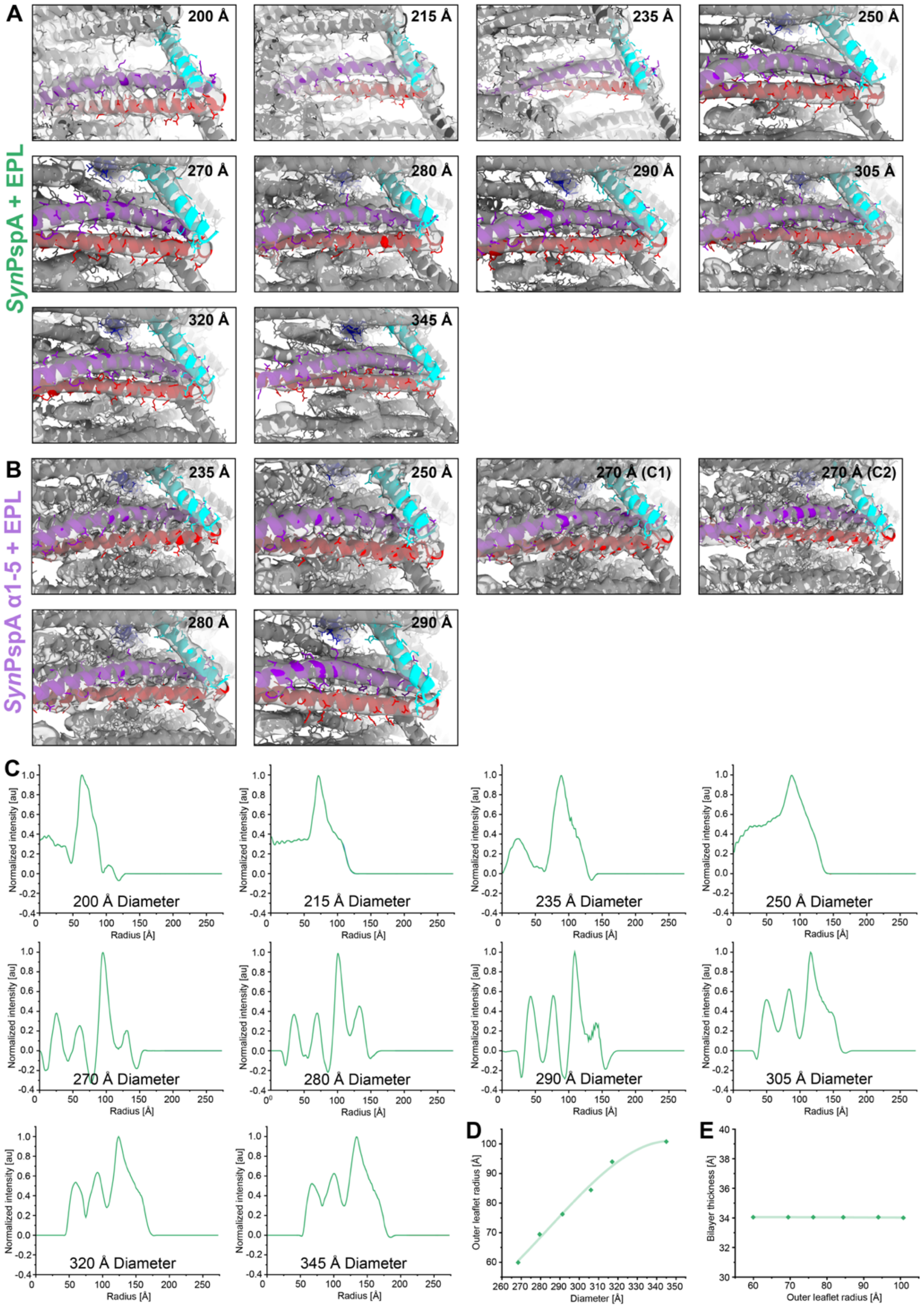

Figure S5: PspA rod density, radial density profiles and mass per length.

A: Density of PspA+EPL assemblies of different diameter rods with the atomic model of the monomer in ribbon representation (α1 red, α2+3 violet, α4 blue, α5 cyan). **B**: Density of PspA α1-5 +EPL assemblies of different diameter rods with the atomic model of the monomer in ribbon representation (α1 red, α2+3 violet, α4 blue, α5 cyan). **C**: Radial density profiles of PspA+EPL rods with diameters ranging from 200 - 345 Å showing typical peaks for a lipid bilayer and the protein density. **D**: Scatter plot of the outer leaflet membrane tube radius from PspA+EPL rods, measured as the max/min from the density profile against the respective bilayer thickness. **E**: Scatter plot of PspA+EPL rods diameters containing significant tubular membrane density against the respective outer leaflet radius of the membrane tube.

Supplement Figure 6

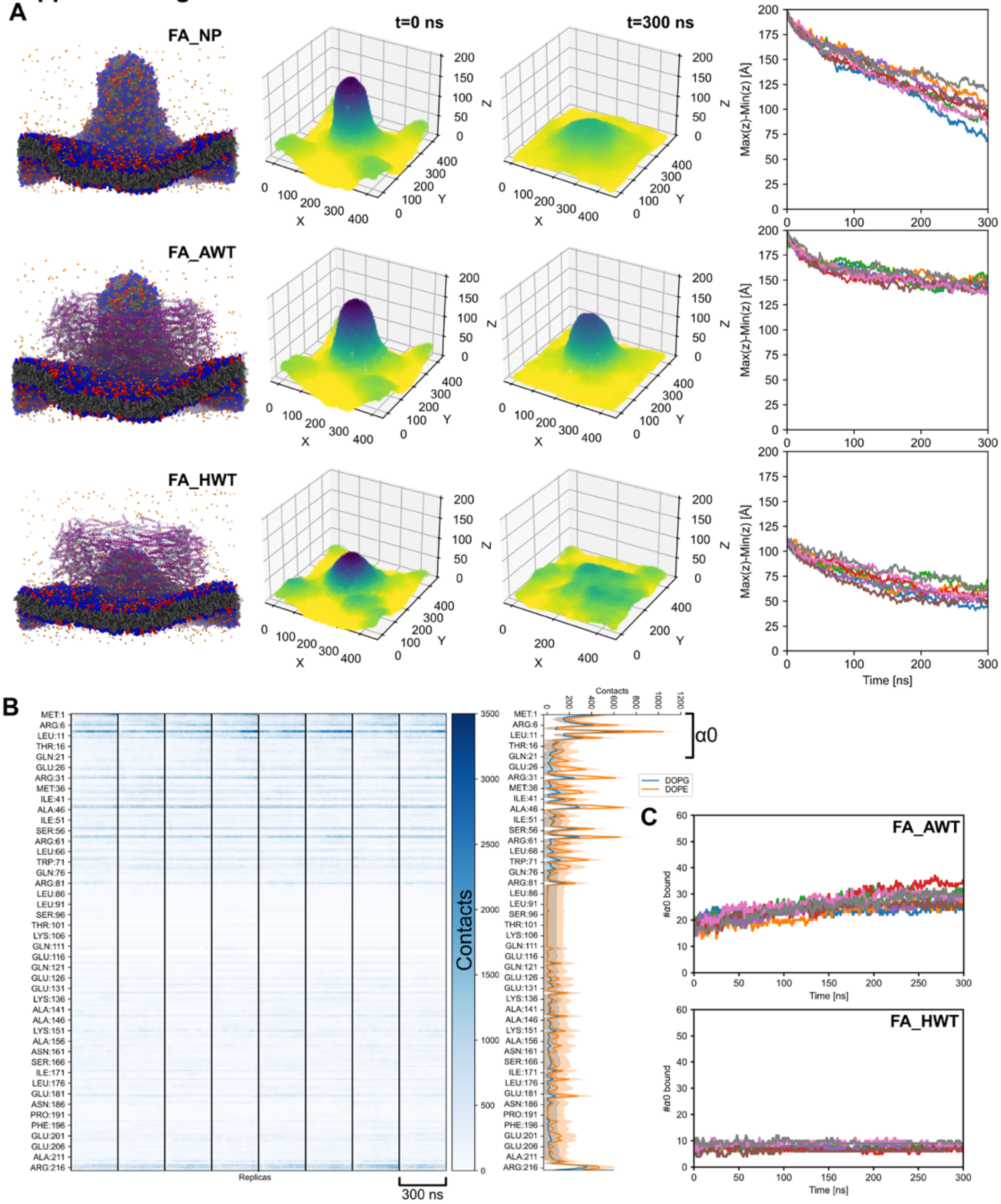

Figure S6: The 290 Å PspA bilayer complex modeled in the full-atom representation.

A: Left: Depiction of full-atom systems in the absence (FA_NP, top) and the presence of PspA, with the curved membrane spanning fully (FA_AWT, middle) and halfway through (FA_HWT, bottom). Center: Surface depictions of the initial (t = 0 ns) and final (t = 300 ns) structures of the upper leaflet of the membrane. Right: The distance between the highest and lowest point of the membrane along the membrane normal as a proxy of the change in membrane curvature. **B**: Summed per-residue number of contacts over the 60 simulated PspA chains in the 290 Å complex with the lipid headgroups on the membrane surface, over eight replicas. To the right, the mean number of contacts with DOPG and DOPE lipids along the trajectory is given, with the standard deviation as the shaded area. **C**: Total number of helices α0 interacting with the membrane during the simulations for FA_AWT (top) and FA_HWT (bottom).

Supplement Figure 7

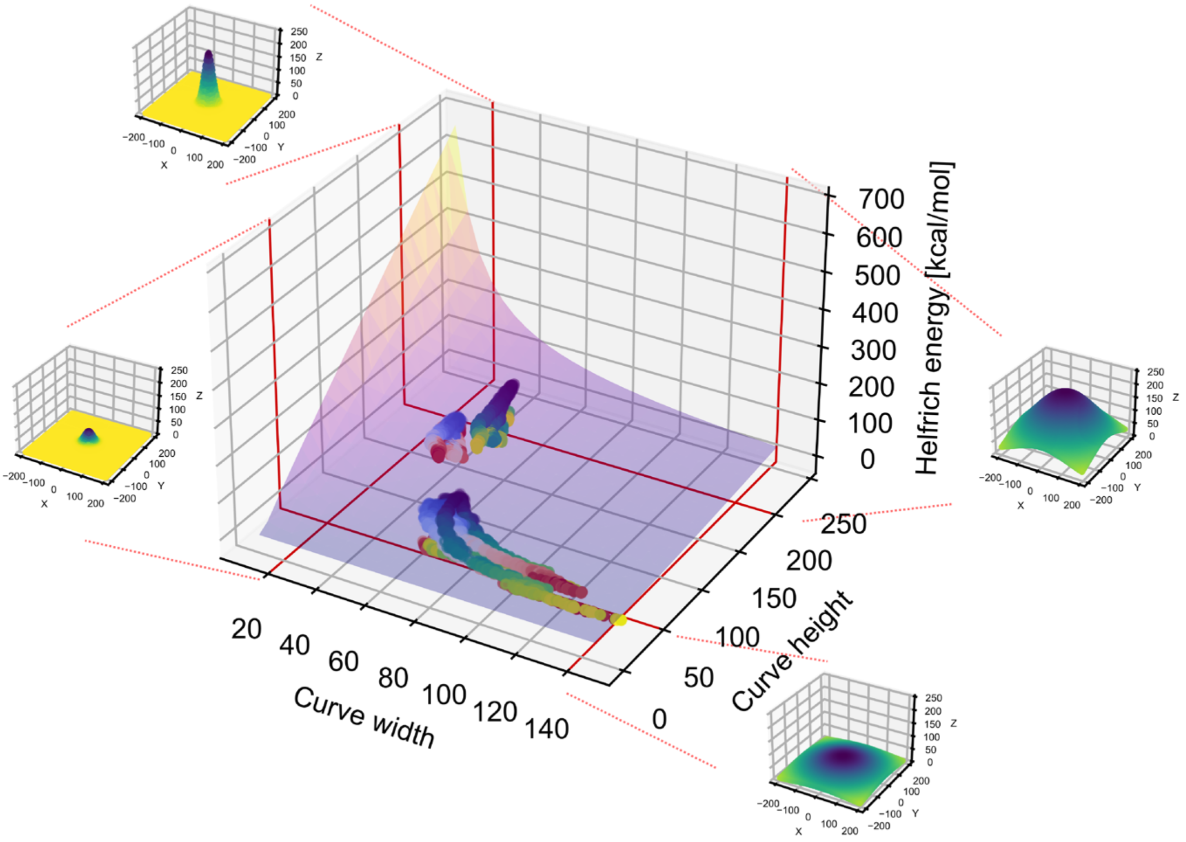

Figure S7: Helfrich bending energy for 2D Gaussians of different amplitudes and standard deviations.

The depicted graph surface is the same as that shown in **Fig. 4D**, here given as a 3D surface rather than a contour plot. Additional Gaussian surfaces for low/high width and height are included as visual aids. Helfrich energies of snapshots of full-atom all-way-through (FA_AWT) and full-atom half-way-through (FA_HWT) trajectories are projected onto the energy surface (blue to yellow, for start to finish upper leaflet, and light blue to red, for start to finish lower leaflet). For further details, refer to the caption of **Fig. 4D**

Supplemental Figure 8

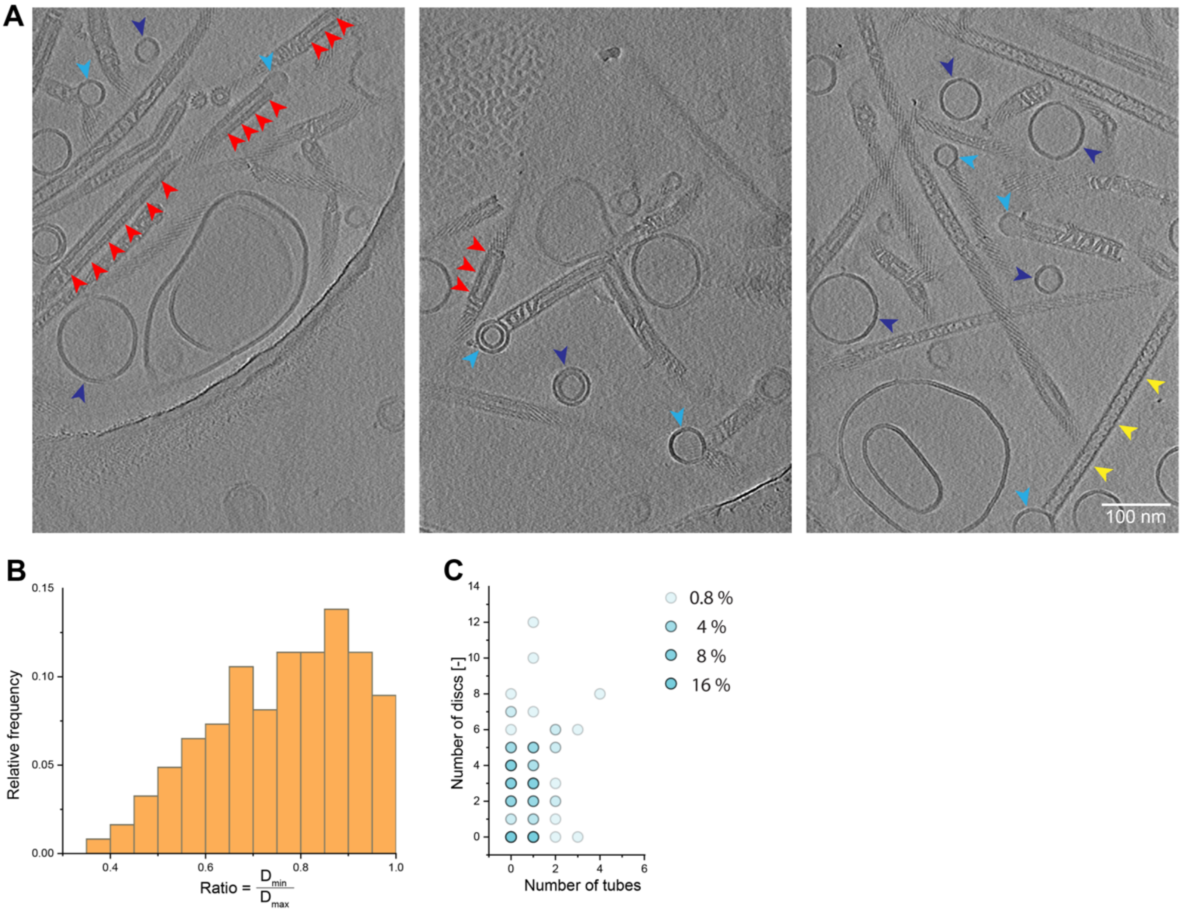

Figure S8: Tomograms of PspA with EPL membranes.

A: Tomogram images of PspA with EPL membranes. Red arrows: completely tubulated EPL vesicles within the PspA rod structures, dark blue arrows: stand-alone vesicles, light blue arrows: rod-attached vesicles, red arrows: rod-internalized vesicles, yellow arrows: PspA rod without EPL membrane in the rod lumen. B: Histogram of the diameter variation per rod, plotted as the ratio of the minimum and maximum diameter within a rod (n = 125). C: Dependency distribution of the membrane structures within the rods (n = 125).

References

1. B. Junglas, *et al.*, PspA adopts an ESCRT-III-like fold and remodels bacterial membranes. *Cell* **184**, 3674-3688.e18 (2021).

2. G. Jovanovic, *et al.*, The N-terminal amphipathic helices determine regulatory and effector functions of phage shock protein A (PspA) in escherichia coli. *Journal of Molecular Biology* **426**, 1498–1511 (2014).

3. C. McDonald, G. Jovanovic, O. Ces, M. Buck, Membrane stored curvature elastic stress modulates recruitment of maintenance proteins pspa and vipp1. *mBio* **6**, e01188-15 (2015).

4. A. Micsonai, *et al.*, BeStSel: webserver for secondary structure and fold prediction for protein CD spectroscopy. *Nucleic Acids Research* **50**, W90–W98 (2022).

5. S. Ahmad, *et al.*, Substrate Access Mechanism in a Novel Membrane-Bound Phospholipase A of *Pseudomonas aeruginosa* Concordant with Specificity and Regioselectivity. *J. Chem. Inf. Model.* **61**, 5626–5643 (2021).

6. H. R. Bureau, D. R. Merz, E. Hershkovits, S. Quirk, R. Hernandez, Constrained Unfolding of a Helical Peptide: Implicit versus Explicit Solvents. *PLoS ONE* **10**, e0127034 (2015).

7. A. Grossfield, WHAM: the weighted histogram analysis method. (2024). Available at: http://membrane.urmc.rochester.edu/wordpress/?page_id=126.

8. N. Ben-Tal, B. Honig, C. K. Bagdassarian, A. Ben-Shaul, Association Entropy in Adsorption Processes. *Biophysical Journal* **79**, 1180–1187 (2000).

9. I. Y. Ben-Shalom, S. Pfeiffer-Marek, K.-H. Baringhaus, H. Gohlke, Efficient Approximation of Ligand Rotational and Translational Entropy Changes upon Binding for Use in MM-PBSA Calculations. *J. Chem. Inf. Model.* **57**, 170–189 (2017).

10. A. V. Finkelstein, J. Janin, The price of lost freedom: entropy of bimolecular complex formation. *Protein Eng Des Sel* **3**, 1–3 (1989).

11. E. E. Moutoussamy, *et al.*, Standard Binding Free Energy and Membrane Desorption Mechanism for a Phospholipase C. *J. Chem. Inf. Model.* **62**, 6602–6613 (2022).

12. A. Ben-Shaul, N. Ben-Tal, B. Honig, Statistical thermodynamic analysis of peptide and protein insertion into lipid membranes. *Biophysical Journal* **71**, 130–137 (1996).

13. F. Fogolari, O. Maloku, C. J. Dongmo Foumthuim, A. Corazza, G. Esposito, PDB2ENTROPY and PDB2TRENT: Conformational and Translational–Rotational Entropy from Molecular Ensembles. *J. Chem. Inf. Model.* **58**, 1319–1324 (2018).

14. M. R. Machado, S. Pantano, SIRAH tools: mapping, backmapping and visualization of coarse-grained models. *Bioinformatics* **32**, 1568–1570 (2016).

15. L. Martínez, R. Andrade, E. G. Birgin, J. M. Martínez, P ACKMOL : A package for building initial configurations for molecular dynamics simulations. *J Comput Chem* **30**, 2157–2164 (2009).

16. L. Darré, M. R. Machado, P. D. Dans, F. E. Herrera, S. Pantano, Another Coarse Grain Model for Aqueous Solvation: WAT FOUR? *J. Chem. Theory Comput.* **6**, 3793–3807 (2010).

17. W. Helfrich, Elastic Properties of Lipid Bilayers: Theory and Possible Experiments. *Zeitschrift für Naturforschung C* **28**, 693–703 (1973).

18. M. Hu, J. J. Briguglio, M. Deserno, Determining the Gaussian Curvature Modulus of Lipid Membranes in Simulations. *Biophysical Journal* **102**, 1403–1410 (2012).

19. J. F. Nagle, Introductory Lecture: Basic quantities in model biomembranes. *Faraday Discuss.* **161**, 11–29 (2013).

20. Z. Chen, R. P. Rand, Comparative Study of the Effects of Several n-Alkanes on Phospholipid Hexagonal Phases. *Biophysical Journal* **74**, 944–952 (1998).

21. H. Bhatia, H. I. Ingólfsson, T. S. Carpenter, F. C. Lightstone, P.-T. Bremer, MemSurfer: A Tool for Robust Computation and Characterization of Curved Membranes. *J. Chem. Theory Comput.* **15**, 6411–6421 (2019).

22. W. Schroeder, K. Martin, B. Lorensen, *The Visualization Toolkit* (2018).

23. A. G. Megrabov, On divergence representations of the Gaussian and the mean curvature of surfaces and applications. (2014).

24. M. Trott, *The Mathematica guidebook for graphics* (2004).

25. D. Tegunov, P. Cramer, Real-time cryo-electron microscopy data preprocessing with Warp. *Nature Methods* **16**, 1146–1152 (2019).

26. S. Zheng, *et al.*, AreTomo: An integrated software package for automated marker-free, motion-corrected cryo-electron tomographic alignment and reconstruction. *Journal of Structural Biology: X* **6**, 100068 (2022).

27. E. F. Pettersen, *et al.*, UCSF ChimeraX: Structure visualization for researchers, educators, and developers. *Protein Sci* **30**, 70–82 (2021).

28. C. T. Rueden, *et al.*, ImageJ2: ImageJ for the next generation of scientific image data. *BMC Bioinformatics* **18**, 529 (2017).

29. D. R. Roe, T. E. Cheatham, PTRAJ and CPPTRAJ: Software for Processing and Analysis of Molecular Dynamics Trajectory Data. *J. Chem. Theory Comput.* **9**, 3084–3095 (2013).

30. W. DeLano, PyMOL. *DeLano Scientific, San Carlos, CA* 1–15 (2002).

31. Schrödinger, The PyMOL Molecular Graphics System.

32. W. Humphrey, A. Dalke, K. Schulten, VMD: Visual molecular dynamics. *Journal of Molecular Graphics* **14**, 33–38 (1996).

33. C. R. Harris, *et al.*, Array programming with NumPy. *Nature* **585**, 357–362 (2020).

34. P. Virtanen, *et al.*, SciPy 1.0: fundamental algorithms for scientific computing in Python. *Nat Methods* **17**, 261–272 (2020).

35. A. Meurer, *et al.*, SymPy: symbolic computing in Python. *PeerJ Computer Science* **3**, e103 (2017).

36. C. Sun, B. Gonzalez, W. Jiang, Helical Indexing in Real Space. *Sci Rep* **12**, 8162 (2022).

37. X. Zhang, Python-based Helix Indexer: A graphical user interface program for finding symmetry of helical assembly through Fourier-Bessel indexing of electron microscopic data. *Protein Sci* **31**, 107–117 (2022).
